# Donor myeloid derived suppressor cells (MDSCs) prolong allogeneic cardiac graft survival through programming of recipient myeloid cells in vivo

**DOI:** 10.1038/s41598-020-71289-z

**Published:** 2020-08-28

**Authors:** Songjie Cai, John Y. Choi, Thiago J. Borges, Hengcheng Zhang, Ji Miao, Takaharu Ichimura, Xiaofei Li, Simiao Xu, Philip Chu, Siawosh K. Eskandari, Hazim Allos, Juliano B. Alhaddad, Saif A. Muhsin, Karim Yatim, Leonardo V. Riella, Peter T. Sage, Anil K. Chandraker, Jamil R. Azzi

**Affiliations:** 1 Renal Division, Transplantation Research Center, Brigham and Women’s Hospital, Harvard Medical School, 221 Longwood Ave, Boston, MA 02115 USA; 2 Division of Endocrinology, Boston Children’s Hospital, Harvard Medical School, Boston, MA USA

**Keywords:** Immunotherapy, Transplant immunology

## Abstract

Solid organ transplantation is a lifesaving therapy for patients with end-organ disease. Current immunosuppression protocols are not designed to target antigen-specific alloimmunity and are uncapable of preventing chronic allograft injury. As myeloid-derived suppressor cells (MDSCs) are potent immunoregulatory cells, we tested whether donor-derived MDSCs can protect heart transplant allografts in an antigen-specific manner. C57BL/6 (H2K^b^, I-A^b^) recipients pre-treated with BALB/c MDSCs were transplanted with either donor-type (BALB/c, H2K^d^, I-A^d^) or third-party (C3H, H2K^k^, I-A^k^) cardiac grafts. Spleens and allografts from C57BL/6 recipients were harvested for immune phenotyping, transcriptomic profiling and functional assays. Single injection of donor-derived MDSCs significantly prolonged the fully MHC mismatched allogeneic cardiac graft survival in a donor-specific fashion. Transcriptomic analysis of allografts harvested from donor-derived MDSCs treated recipients showed down-regulated proinflammatory cytokines. Immune phenotyping showed that the donor MDSCs administration suppressed effector T cells in recipients. Interestingly, significant increase in recipient endogenous CD11b^+^Gr1^+^ MDSC population was observed in the group treated with donor-derived MDSCs compared to the control groups. Depletion of this endogenous MDSCs with anti-Gr1 antibody reversed donor MDSCs-mediated allograft protection. Furthermore, we observed that the allogeneic mixed lymphocytes reaction was suppressed in the presence of CD11b^+^Gr1^+^ MDSCs in a donor-specific manner. Donor-derived MDSCs prolong cardiac allograft survival in a donor-specific manner via induction of recipient’s endogenous MDSCs.

## Introduction

Solid organ transplantation is a lifesaving therapy for patients with end-organ disease. The Scientific Registry of Transplant Recipients (SRTR) reported that 14,313 kidney^1^, 7,483 liver^2^, 2,465 lung^3^, 432 heart^4^, 80 pancreas^5^, and 109 intestinal^6^ transplants were performed in 2017 in the US (Feb 2019 updated). Nevertheless, number of challenges remain for organ recipients after the transplantation, as they suffer from acute and chronic rejection as well as complications of immunosuppression^7–9^. One of the more critical issues pertains to the current state of standard immunosuppressive protocols. Calcineurin inhibitor (CNI) based immunosuppressive protocols are not designed to promote antigen-specific tolerance, and may exacerbate chronic allograft injury (i.e. calcineurin toxicity)^10^. Therefore, there is an urgent need to establish safer anti-rejection strategies by targeting antigen-specific alloimmunity.

Cell therapies, including regulatory dendritic cells (DCregs)^11–14^, regulatory T cells (Tregs)^15^ and regulatory B cells (Bregs)^16^, have shown the potential application in solid organ transplantation. In particular, Thompson and colleagues conducted a first-in-human study to demonstrate that adoptive transfer of DCreg allows complete withdrawal of immunosuppression regimen in low risk patients^17–20^. Myeloid derived suppressor cells (MDSCs) comprise a heterogeneous cell population including immature myeloid cells, which thereafter differentiate into monocytes, dendritic cells and neutrophils^21^. While sharing similar functional features, MDSCs are shown to offer much powerful immunosuppression compared to DCregs. MDSCs inhibit T cell responses by inducing the apoptosis of antigen-primed and activated T cells^22,23^ while inducing FoxP3^+^ Treg^23,24^. In vivo models have shown MDSCs attenuate GvHD^25^ as well as autoimmunity^26,27^. Finally, higher frequency of MDSCs were detected in blood samples from stable renal transplant patients^24^.

Based on the above clinical and preclinical studies, we hypothesized that donor-derived MDSCs may induce donor-specific immune suppression in allogeneic organ transplantation. Of note, we modified standard in vitro MDSCs generation protocol^28^ by culturing donor bone marrow cells within 6 days in the presence of GM-CSF^29^ , TGFβ^30^ and IL10^31,32^. In addition, we added IFNγ on day 5 to promote the suppressive function of MDSCs^33^. Finally, we demonstrated that the administration of donor-derived MDSCs prolong allogeneic cardiac graft survival in a donor-specific manner through induction of recipients’ endogenous MDSCs.

## Results

### *Donor-derived MDSCs suppress alloreactive T cell activation *in vitro

**Figure 1:**
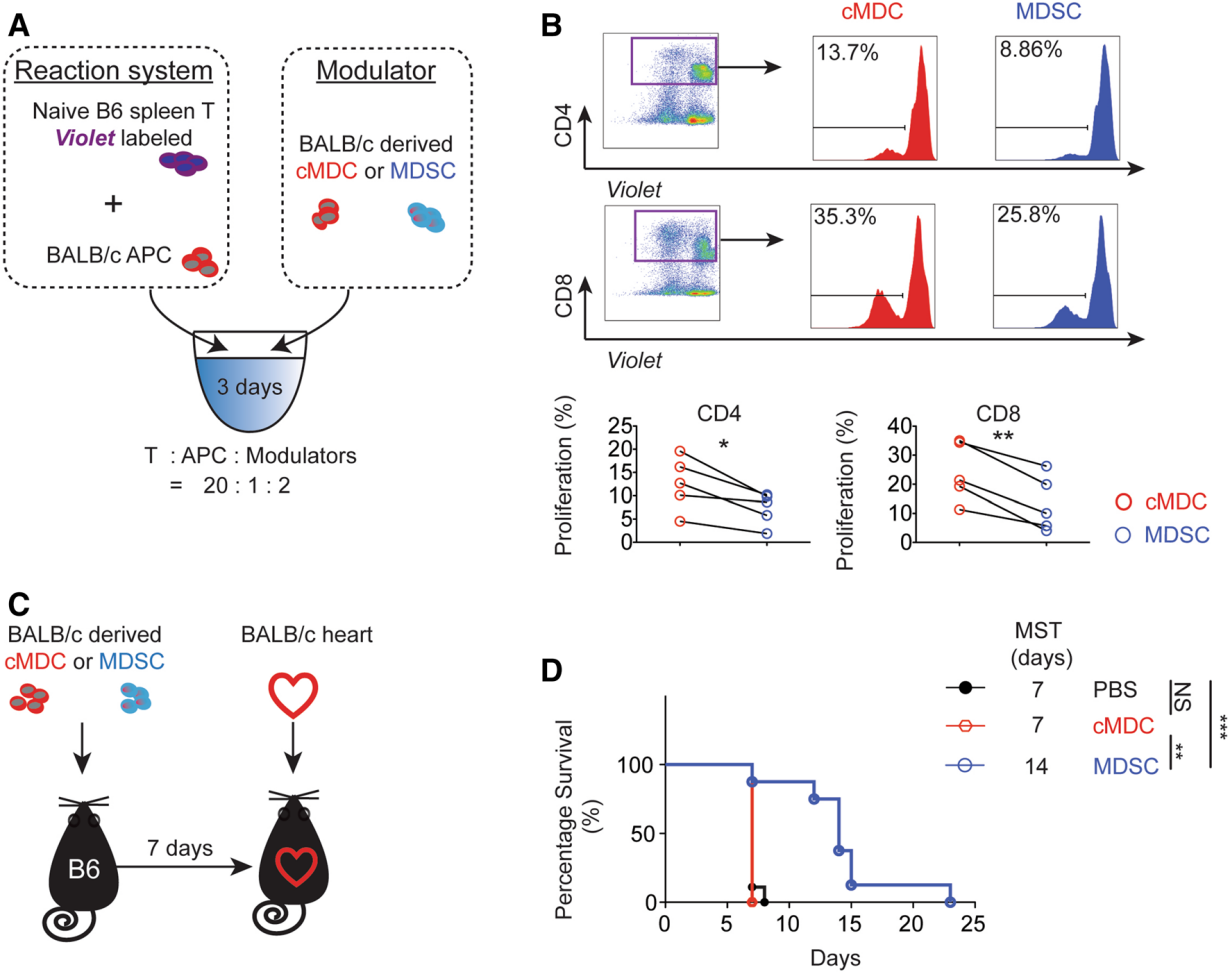
Donor-derived MDSCs suppress alloimmune reaction in vitro and in vivo. (**A**) Naïve C57BL/6T cells were stimulated with BALB/c antigen presenting cells (APCs: BALB/c bone marrow derived cDCs). MDSCs or cMDCs were added as modulator. CD4^+^ and CD8^+^ T cell proliferation in response to allogeneic cDCs was analyzed by CellTrace *violet* dye dilution (n = 5 per group). (**B**) Graphs showing the proliferation of CD4^+^ and CD8^+^ T cells in the presence of MDSCs compared to cMDCs. Mean ± SEM, *p < 0.05, **p < 0.01, two-tailed unpaired t test. Data represents one of 4 separate experiments. (**C**) Schematic diagram of the experimental design. C57BL/6 recipients received a single-dose intravenous injection of 1 × 10^6^ BALB/c MDSCs or cMDCs 7 days prior to the cardiac transplantation. (**D**) Kaplan–Meier cumulative survival of allograft shows the prolonged survival in MDSCs treated group compared to control groups (n = 8–9 per group). **p < 0.01, ***p < 0.001, log-rank test.

Allogeneic mixed lymphocyte reaction (alloMLR) was performed to examine the regulatory function of donor-derived MDSCs. Co-culturing naive T cells isolated from C57BL/6 mice (H2K^b^, I-A^b^) with BALB/c (H2K^d^, I-A^d^) derived conventional DCs (cDCs) stimulates an alloreactive T cell proliferation. We observed that the addition of BALB/c MDSCs to this alloMLR system significantly inhibited the proliferation of CD4^+^ and CD8^+^ T cells compared to conventional myeloid derived cells (cMDCs) (Fig. 1A,B).

### Donor-derived MDSCs protect cardiac allografts from acute rejection

We then examined the in vivo suppressive function of donor-derived MDSCs in the allogeneic cardiac transplantation model. C57BL/6 recipients received a single-dose of intravenous injection of 1 × 10^6^ BALB/c MDSCs or BALB/c cMDCs 7 days prior to the cardiac transplantation with BALB/c donors (Fig. 1C). We found that BALB/c MDSCs significantly prolonged allograft survival; in contrast, administration of cMDCs showed no difference to the group receiving PBS (PBS control n = 9, median survival time (MST) 7 days; BALB/c cMDCs control n = 7, MST 7 days; BALB/c MDSCs n = 8, MST 14 days, Fig. 1D).

**Figure 2:**
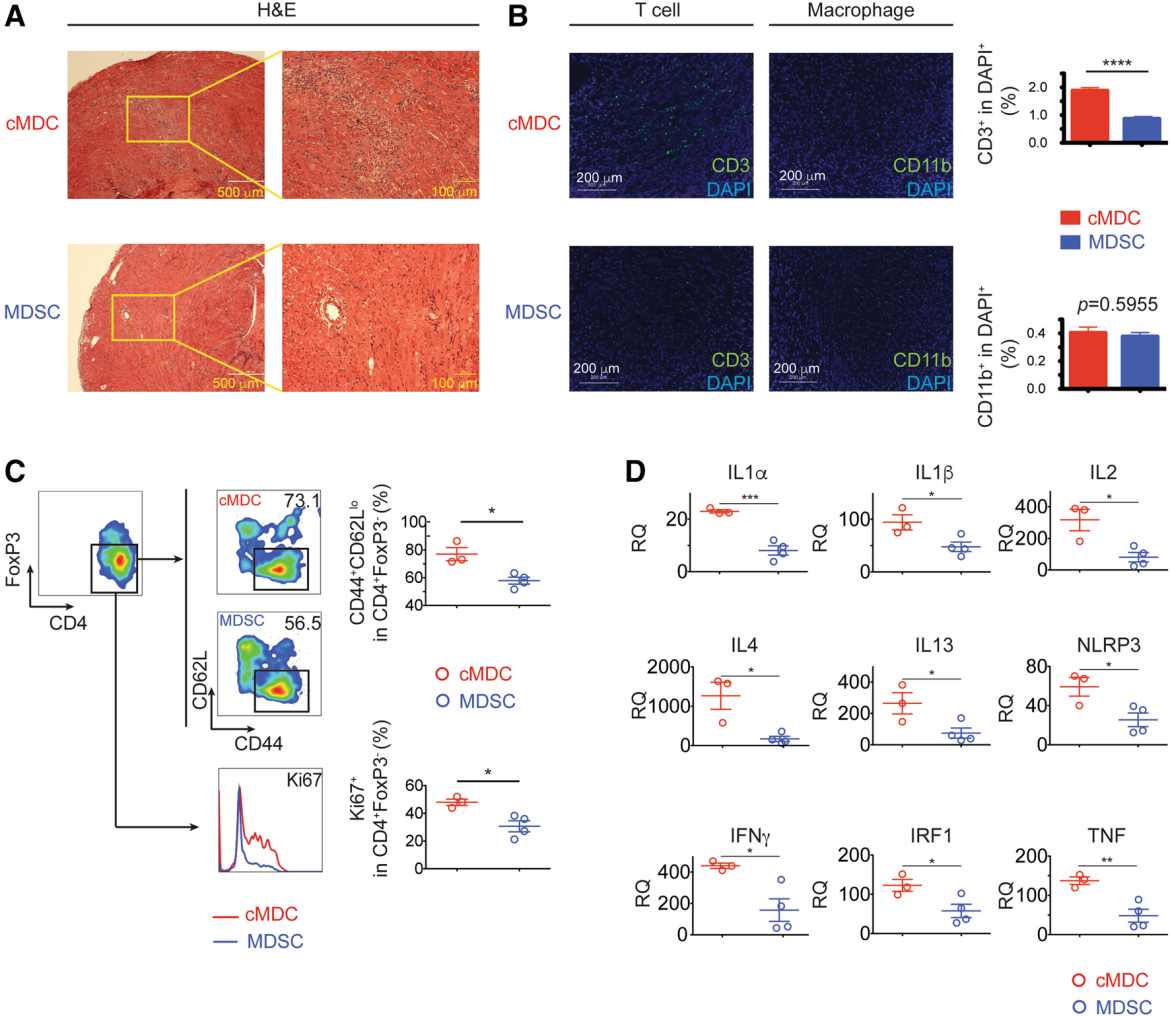
Allografts from donor-derived MDSCs treated recipients exhibit ameliorated T cell mediated inflammation and increased recipient-derived endogenous MDSCs. (**A**) Representative example of the allograft histology (H&E staining) from the MDSCs treated group and the cMDCs treated control on POD7. Scale bar represent 500 μm (left) and 100 μm (right). Data represents the one of 3 separate experiments. (**B**) Allografts were harvested on POD7 and stained with anti-CD3, anti-CD11b (green) and DAPI (blue). Scale bars represent 200 μm. Positive cells were qualitied by ImageJ and expressed as mean ± SEM. ****p < 0.0001, two-tailed unpaired t test. (**C**) Flow cytometry analysis of BALB/c MDSCs treated C57BL/6 recipients’ graft infiltration lymphocytes (GILs) on POD7. Graphs showing significant decrease of the proportion of CD44^+^CD62L^lo^ and Ki67^+^ in CD4^+^FoxP3^-^ T cells in the MDSCs treated group. (**D**) qRT-PCR analysis of allografts (whole tissues) on POD3 (n = 3 per group). Graph represented as RQ (relative quantification) = 2^-ΔΔCt^. Naïve BALB/c heart serve as basic ΔCt (n = 3, not shown in graph). Mean ± SEM, *p < 0.05, **p < 0.01, ***p < 0.001, two-tailed unpaired t test.

Allografts were harvested on postoperative day (POD) 7 for hematoxylin and eosin (H&E) staining and immune fluorescence staining. H&E staining revealed the attenuated myocardial lesion as well as the decreased lymphocyte infiltration in the donor MDSCs treated group compared to the cMDC group (Fig. 2A). Immune fluorescence staining showed that CD3^+^ T cells were significantly decreased in the MDSC group compared to the cMDC group. Of note, there was no difference in CD11b^+^ cells infiltration between two groups (Fig. 2B).

Graft infiltration lymphocytes (GILs) were isolated from allografts (POD7) for flow cytometry analysis. Gating on the CD4^+^FoxP3^-^ helper T cells, the proportion of effector T cells (Teff) defined as CD44^+^CD62L^lo^ population, was significantly decreased in the MDSCs treated group compared to the cMDCs treated group (Fig. 2C-upper). Within this Teff, the activation level measured by Ki67 was also significantly reduced in the MDSCs treated group (Fig. 2C-lower).

Finally, qRT-PCR of allografts (POD3, whole tissue) showed that the treatment with donor-derived MDSCs markedly downregulated the mRNA expression of proinflammatory cytokine: IL1α, IL2, IL4, IL13, NLRP3, IFNγ, IRF1, and TNF (Fig. 2D and Table S4).

### Allo-immune response of effector T cells is reduced in recipients treated with donor MDSCs

**Figure 3:**
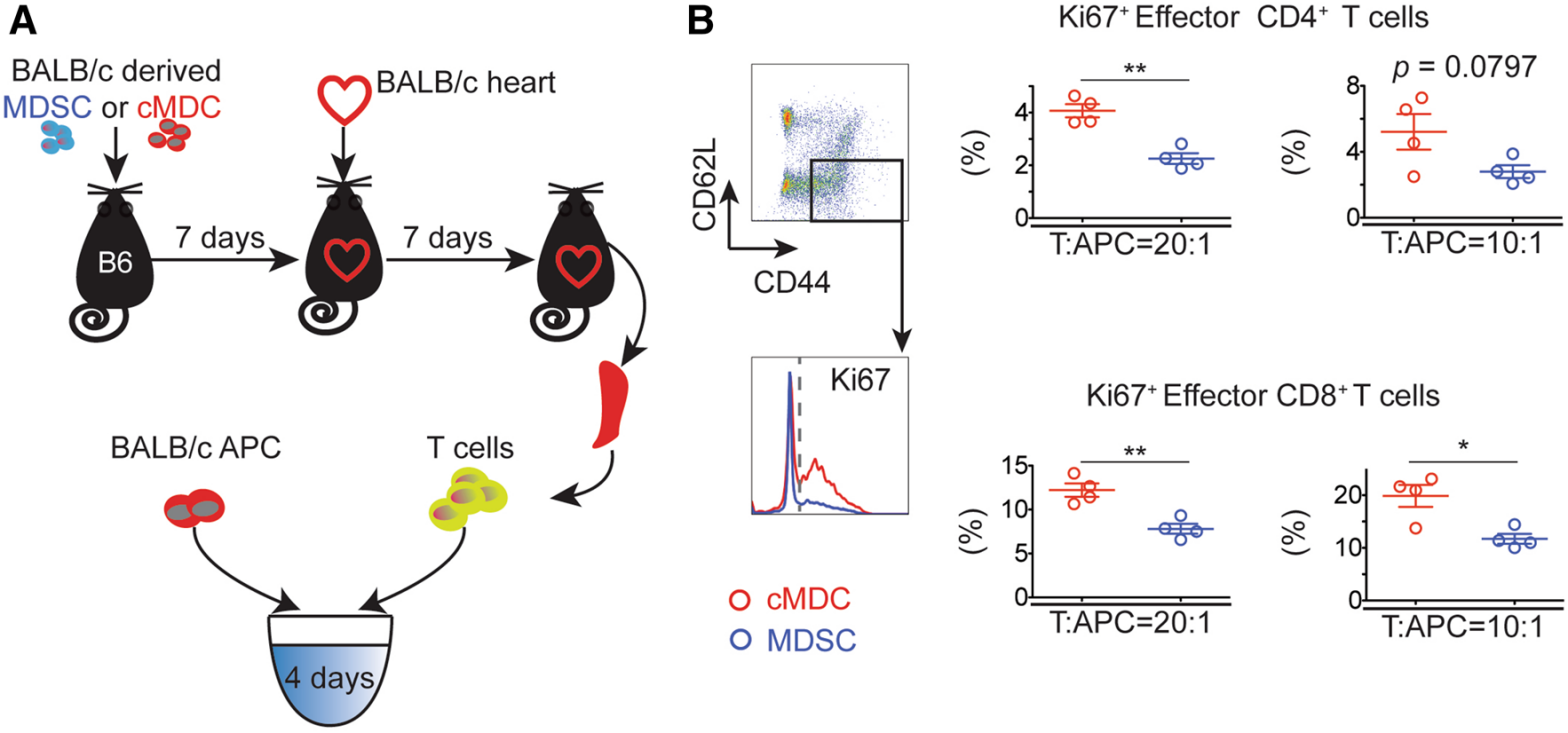
Donor-derived MDSCs suppress effector T cell activation. (**A**) Schematic diagram of the experimental design. T cells were isolated from recipient splenocytes on POD7 and were stimulated with BALB/c cDCs for 4 days (n = 4 per group). (**B**) CD4^+^ and CD8^+^ effector T cells induction was analyzed by flow cytometry. Cells were gated on CD4^+^ Teff (CD4^+^FoxP3^-^CD44^+^CD62^lo^) and CD8^+^ Teff (CD8^+^FoxP3^-^CD44^+^CD62^lo^). Graphs showing the percentage of activated Teff (Ki67^+^Teff). Mean ± SEM, *p < 0.05, **p < 0.01, two-tailed unpaired t test. Data represents one of 4 separate experiments.

T cells were isolated from recipients’ splenocytes on POD7 for the ex vivo stimulation assay using BALB/c Antigen Presenting Cells (APCs) at the ratio of 20:1 or 10:1 for 4 days (Fig. 3A). CD4^+^ and CD8^+^ Teff induction was analyzed by flow cytometry. Cells were gated on CD4^+^ Teff (CD4^+^FoxP3^-^CD44^+^CD62^lo^) and CD8^+^ Teff (CD8^+^FoxP3^-^CD44^+^CD62^lo^). We found that the proportion of Ki67^+^ Teff (Ki67^+^CD44^+^CD62^lo^) in CD4^+^FoxP3^-^ as well as CD8^+^FoxP3^-^ population were decreased in the MDSCs group in comparison to the cMDCs group (Fig. 3B).

### Donor-derived MDSCs induce highly immune suppressive endogenous MDSCs

**Figure 4:**
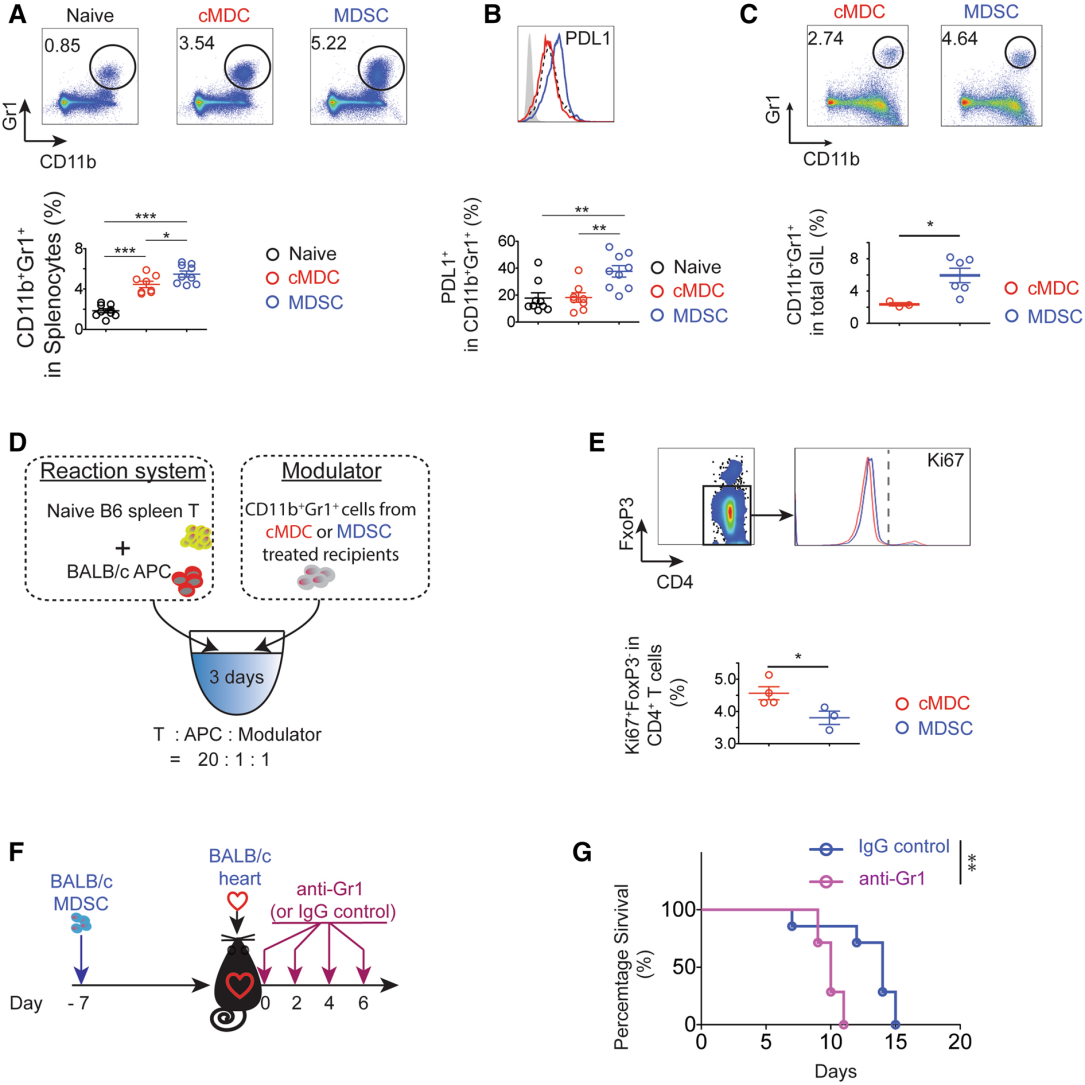
Endogenous MDSCs (CD11b^+^Gr1^+^) are essential to donor-derived MDSCs mediated alloimmune suppression. (**A**,**B**) Flow cytometry analysis of BALB/c MDSCs treated C57BL/6 recipients’ splenocytes on POD7 (n = 8–9 per group). (**A**) The proportion of CD11b^+^Gr1^+^ significantly increased in donor-derived the MDSCs treated group. (**B**) Cells were gated on CD11b^+^Gr1^+^, PDL1 expression was up-regulated in the MDSCs treated group. Mean ± SEM, * p < 0.05, ** p < 0.01, *** p < 0.001, one-way ANOVA and Tukey’s test. Data represents one of 4 separate experiments. (**C**) Flow cytometry analysis of BALB/c MDSCs treated C57BL/6 recipients’ graft infiltration lymphocytes (GILs) on POD7. CD11b^+^Gr1^+^ population was significantly increased in MDSCs treated group. n = 3–6 per group. Mean ± SEM. Data represents one of 3 separate experiments. *p < 0.05, two-tailed unpaired t test. (**D**) Schematic illustration of the experimental design to test the ex vivo immunosuppressive function of BALB/c MDSCs induced endogenous MDSCs (CD11b^+^Gr1^+^) in recipients. Naïve C57BL/6 T cells were stimulated with BALB/c APCs for 3 days. CD11b^+^Gr1^+^ cells were isolated by fluorescence activated cell sorting (FACS) from C57BL/6 recipient splenocytes at POD7 (n = 3–4 per group) and were added as modulator. (**E**) Cells were gated on CD4^+^FoxP3^-^, and the Ki67 expression was measured. Graphs showing significant decrease of the proportion of Ki67^+^FoxP3^-^ in CD4^+^ T cells in the presence of CD11b^+^Gr1^+^ cells induced with MDSCs compared to those induced with cMDCs. Mean ± SEM, * p < 0.05, two-tailed unpaired t test. Data represents one of 3 separate experiments. (**F**) Schematic illustration of the treatment protocol. C57BL/6 recipients received a single-dose intravenous injection of 1 × 10^6^ BALB/c 7 days prior to the transplantation. Recipients were treated with anti-Gr1 mAb on POD 0, 2, 4, 6. (**G**) Anti-Gr1 mAb administration attenuates MDSCs induced immune suppression. Kaplan–Meier cumulative survival of allograft showing the shortened allograft survival in the anti-Gr1 Ab treated group compared to the IgG treated control group (n = 7 per group). **p < 0.01, log-rank test.

For mechanistic studies, spleens from C57BL/6 recipients of BALB/c hearts treated with BALB/c MDSCs were isolated on POD7 for flow cytometry analysis. We observed that a suppressive population CD11b^+^Gr1^+^^34^, defined broadly as MDSCs^34^, was significantly increased in the donor-derived MDSCs treated group (Fig. 4A). Gating on this population, we found that the PDL1 expression was markedly up-regulated in the MDSCs treated group compared to the cMDCs treated group (Fig. 4B). These data indicate that the donor-derived MDSCs administration not only increases the number of CD11b^+^Gr1^+^ population in recipients, but also program CD11b^+^Gr1^+^ cells to upregulate PDL1 expression. PD-L1/PD-1 interactions were reported to deliver co-inhibitory signals leading to attenuation of T cell responses both in vitro and in vivo^35,36^. Furthermore, PDL1 has been shown to be required for suppression of the autoimmune responses^37^. In consistent with this, we observed significantly increased CD11b^+^Gr1^+^ population in GILs from recipients treated with donor-derived MDSCs (Fig. 4C). We then hypothesized that CD11b^+^Gr1^+^ cells in recipients detected upon administration of donor-derived MDSCs play a crucial role in allogeneic immune suppression.

To determine whether the increase of MDSCs (CD11b^+^Gr1^+^) is from the clonal expansion of the transferred donor-derived MDSCs or from the induction of recipients’ endogenous MDSCs, we traced the transferred donor-derived MDSCs using H2K^d^ antibody. 1 × 10^6^ BALB/c MDSCs were intravenously injected to C57BL/6 recipients, and their splenocytes were examined at 3, 6 and 24 h post-injection. We found that the transferred donor-derived MDSCs peaked 3 h post-injection (PIH3) and then disappeared at PIH24 (Fig. S7A). In parallel, we analyzed the induction of endogenous MDSCs in recipients’ spleens by gating on the recipient type MHC-I, H2K^b^. We found significant increase of these endogenous MDSCs in recipients starting at PIH24 (Fig. S7B). Next, we measured the MHC-II expression on the endogenous MDSCs as a marker of suppressive function^38^. Indeed, the MHC-II expression was markedly elevated on the endogenous MDSCs (Fig. S7B). These data suggest that while donor-derived MDSCs disappear within 24 h, the functional endogenous MDSCs are expanded in the recipients’ lymphoid tissue.

We further characterized the composition of endogenous MDSCs in recipient’s spleen by using Ly6C and Ly6G antibodies^21^. Gating on CD11b^+^ cells, flow cytometry analysis indicated that the induced endogenous MDSCs were composed mostly of Ly6C^+^Ly6G^-^ monocytic MDSC (M-MDSC) and Ly6C^+^Ly6G^+^ MDSC (Fig. S2C,D).

Previous studies have shown that systemic administration of donor cells undergoing apoptosis promote donor-specific immunosuppression in vitro^39^ and in vivo^40,41^. To study whether our generated donor MDSCs suppressed alloimmune reaction is related to the apoptotic donor cell mediated suppression, we used 7-AAD and Annexin-V to measure the frequency of apoptotic cells in MDSCs and control cMDCs. Result showed approximately 3.3% early apoptotic and 6.1% late apoptotic cells in generated donor MDSCs, which are significantly lower than in cMDCs (7.0% early apoptotic and 9.9% late apoptotic cells, p < 0.001) (Fig. S5). Taken together, we concluded that the suppressive function of donor-derived MDSCs is independent of apoptotic-cell-induced immune suppression.

### Endogenous MDSCs inhibit effector T cells and prolong allograft survival

We then set up the alloMLR to test the immunomodulatory function of these endogenous MDSCs in vitro. Naive T cells from C57BL/6 mice were stimulated with BALB/c APCs. CD11b^+^Gr1^+^ cells were isolated by fluorescence activated cell sorting (FACS) from recipients’ splenocytes on POD7 and were used as modulators. As the transferred donor-derived MDSCs disappeared 24 h after injection, these CD11b^+^Gr1^+^ MDSCs are endogenous MDSCs (Fig. S7A). Cells were incubated for 3 days and then stained with CD4, FoxP3 and Ki67 for flow cytometry analysis (Fig. 4D). We observed that the proportion of Ki67^+^FoxP3^-^ in CD4^+^ T cells was significantly decreased in the MDSCs group (Fig. 4E).

Next, we depleted this population with a monoclonal antibody (mAb) anti-Gr1 (RB6-8C5) post-transplantation and observed the allograft survival^42–44^. Recipients were treated with 1 × 10^6^ BALB/c MDSCs 7 days prior to the transplantation, followed by the anti-Gr1 injection on days 0, 2, 4 and 6 post-transplantation (Fig. 4F). Gr1^+^ depletion markedly shortened the allograft survival (anti-Gr1 n = 7, MST 10 days; Isotype IgG n = 7, MST 14 days, p = 0.0052; Fig. 4G) confirming that endogenous MDSCs that are characterized by CD11b^+^Gr1^+^ are essential to the allograft protection.

### Endogenous MDSCs protect cardiac allografts in a donor-specific manner

**Figure 5:**
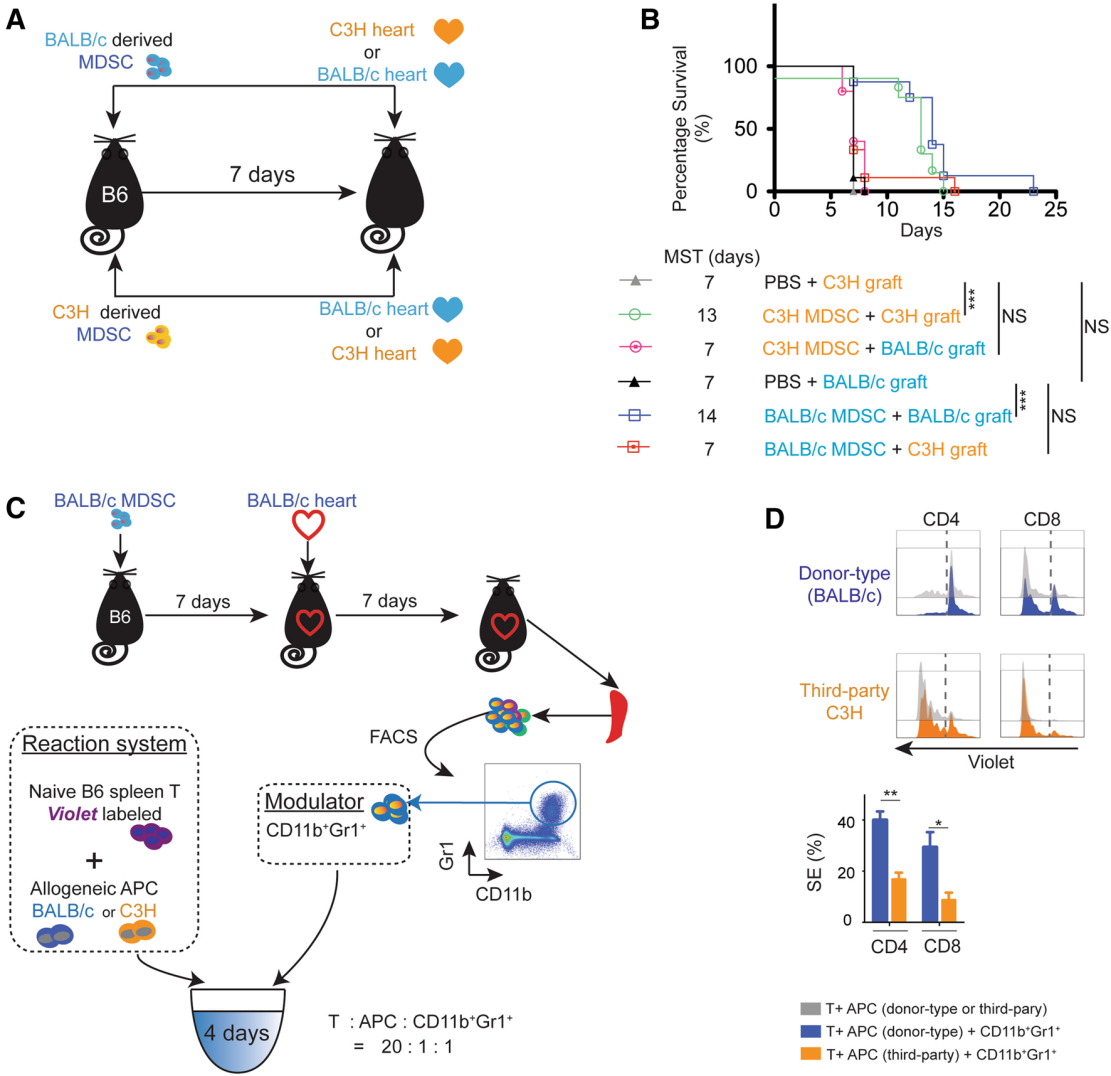
Donor-derived MDSCs prolong allogeneic cardiac graft survival in a donor-specific manner. (**A**) Schematic diagram of the experimental design. C57BL/6 mice received a single-dose intravenous injection of 1 × 10^6^ BALB/c or C3H MDSCs. Cardiac grafts from C3H or BALB/c mice were transplanted into pre-sensitized C57BL/6 recipients. (**B**) Kaplan–Meier cumulative survival of allograft showing that the BALB/c MDSCs administration successfully prolong BALB/c allograft survival but fail to prolong C3H allograft survival, while C3H MDSCs administration prolong C3H allograft survival but fail to prolong BALB/c allograft survival. *p < 0.05, **p < 0.01, ***p < 0.001, log-rank test. (**C**) Schematic illustration of the experimental design to study the e*x vivo* donor-specific immunosuppressive function of donor MDSCs induced endogenous MDSCs (CD11b^+^Gr1^+^). Naïve C57BL/6 spleen T cells were labeled with CellTrace *violet* and were stimulated with BALB/c cDCs or C3H cDCs (third-party). CD11b^+^Gr1^+^ cells were isolated by FACS from BALB/c MDSCs treated C57BL/6 recipient splenocytes at POD7 (n = 3–4 per group) and were added as modulator. (**D**) CD4^+^ and CD8^+^ T cell proliferation in response to primary donor-type BALB/c cDCs or third-party C3H cDCs was analyzed by CellTrace *violet* dye dilution. Graphs showed the attenuated suppression efficiency (SE) of CD11b^+^Gr1^+^ cells in third-party compared to donor-type allo MLR setting. $SE = \frac{{p\left( {w/oMDSC} \right) - p\left( {MDSC} \right)}}{{p\left( {w/oMDSC} \right)}} \times 100\%$. Mean ± SEM, *p < 0.05, **p < 0.01, two-tailed unpaired t test. Data represents one of 3 separate experiments.

We next tested whether the prolongation of the allograft survival by the donor-derived MDSCs administration is donor-specific. C57BL/6 (H2K^b^, I-A^b^) mice received a single-dose intravenous injection of 1 × 10^6^ BALB/c (H2K^d^, I-A^d^) or C3H (H2K^k^, I-A^k^) MDSCs 7 days prior to the transplantation. C3H or BALB/c mice were used as cardiac allograft donors (Fig. 5A). We observed that BALB/c MDSCs was able to prolong BALB/c allograft survival but failed to prolong the C3H allografts survival. Likewise, C3H MDSCs was able to prolong C3H allograft survival but failed to prolong the BALB/c allografts survival (Fig. 5B). Together, this suggests that donor-derived MDSCs prolong the allogeneic cardiac graft survival in a donor-specific manner.

As we have identified that donor-derived MDSCs induced recipient’s endogenous MDSCs (CD11b^+^Gr1^+^) cells in recipients play a key role in allogeneic immune suppression (Fig. 5C,D), we then tested the donor-specific suppressive function of endogenous MDSCs by using the alloMLR. Naïve C57BL/6 spleen T cells were stimulated with BALB/c cDCs or C3H cDCs as a third-party. CD11b^+^Gr1^+^ cells were isolated by FACS from BALB/c MDSCs treated C57BL/6 recipient splenocytes at POD7 and were added as modulators (Fig. 5C). The proliferation of CD4^+^ and CD8^+^ T cells were determined by using *violet* dye dilution by flow cytometry. As naïve C57BL/6 T cells respond to BALB/c and C3H cDCs at a different rate, we calculated the relative suppression efficiency (SE) and compared the SE between the primary donor type cDCs and the third-party cDCs stimulated group (Fig. 5D). We observed doubled SE of CD4^+^ proliferation and a tripled SE of CD8^+^ proliferation in BALB/c cDCs stimulated group compared to C3H cDCs stimulated group (Fig. 5D). This data supports that the immune suppressive function of donor-derived MDSCs induce endogenous MDSCs is antigen-specific.

### *CD11b*^+^*Gr1*^+^*cells among donor-derived MDSCs prolong allograft survival*

**Figure 6:**
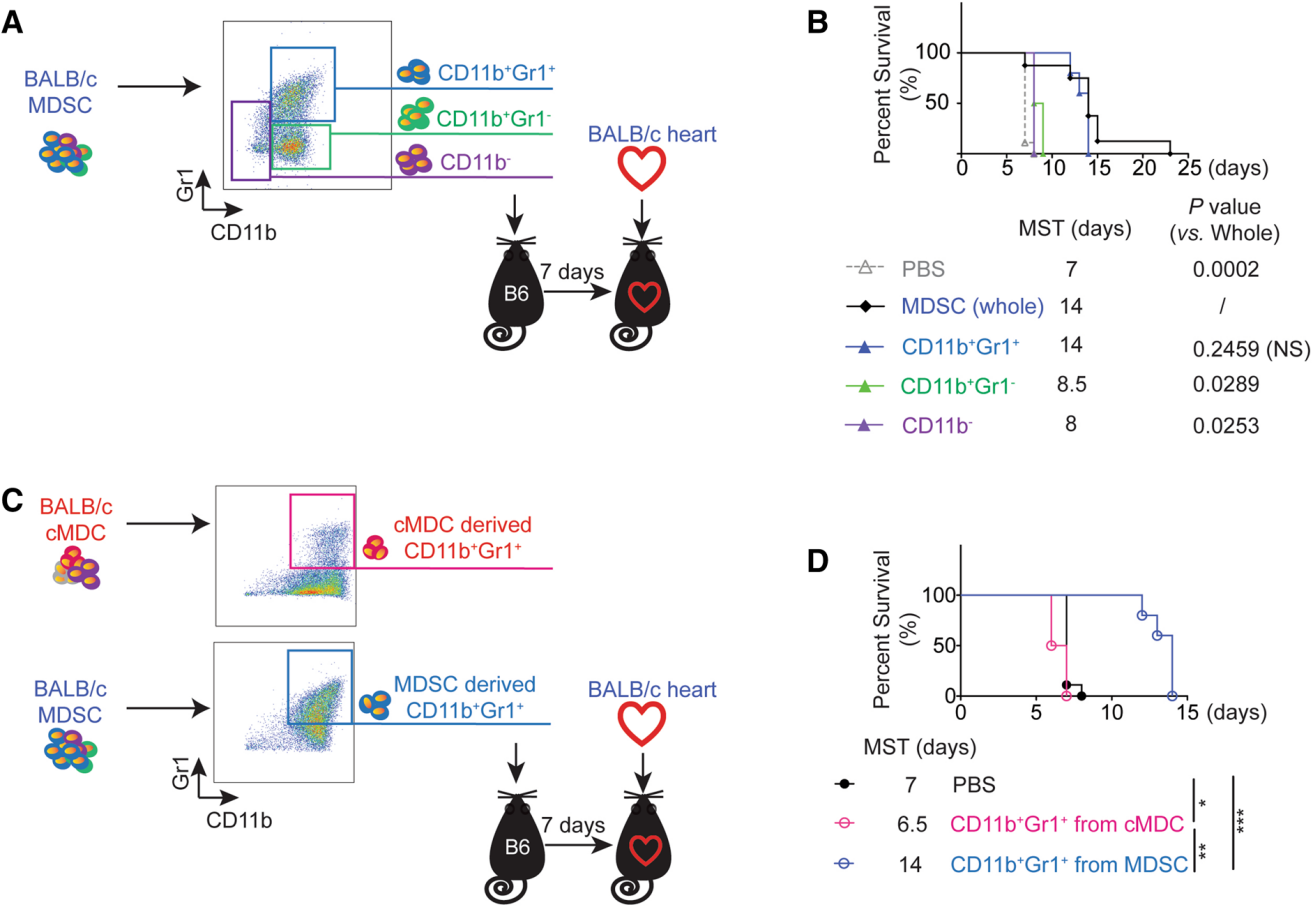
CD11b^+^Gr1^+^ population in donor-derived MDSCs is the effective suppressor subset. (**A**) Schematic illustration of the experimental design to study which subset in donor-derived MDSCs impart the immune suppressive function. C57BL/6 recipients received a single-dose intravenous injection of 1 × 10^6^ CD11b^+^Gr1^+^ or CD11b^+^Gr1^-^ or CD11b^-^ cells FACS isolated from BALB/c derived MDSCs 7 days prior to BALB/c derived cardiac graft transplantation. Whole MDSCs infusion and PBS administration groups served as positive and negative control. (**B**) Kaplan–Meier cumulative survival of allograft showing only CD11b^+^Gr1^+^ subset rather than CD11b^+^Gr1^-^ and CD11b^-^ subsets, reproduces similar allograft prolongation to the whole BALB/c MDSC infusion (p = 0.2459, no significant difference). Log-rank test. (**C**) Schematic diagram of the experimental design to study whether CD11b^+^Gr1^+^ population from MDSCs and cMDCs have the same immune suppressive function. C57BL/6 recipients received a single-dose intravenous injection of 1 × 10^6^ CD11b^+^Gr1^+^ cells FACS isolated from BALB/c MDSC or cMDC 7 days prior to the cardiac transplantation. (**D**) Kaplan–Meier cumulative survival of allograft showing CD11b^+^Gr1^+^ cells from MDSCs prolong allograft survival, while C57BL/6 mice treated with CD11b^+^Gr1^+^ cells from cMDC slightly accelerate the rejection. *p < 0.05, **p < 0.01, ***p < 0.001, log-rank test.

Further analysis of in vitro generated MDSCs revealed the composition of donor-derived MDSCs is > 85% CD11b^+^Gr1^+^ cells and < 15% non-CD11b^+^Gr1^+^ cells (CD11b^-^ and CD11b^+^Gr1^-^) (Fig. S1B). In above experiments pertain to Figs. 1, 2, 3, 4 and 5, we used whole MDSCs that were generated from donor bone marrow cells to achieve the donor-specific alloimmune suppression. To test whether the population other than CD11b^+^Gr1^+^ cells contributes to the biological changes, we isolated the other two populations, CD11b^+^Gr1^-^ and CD11b^-^ cells by FACS and performed immune suppressive functional assay (Fig. 6A).

C57BL/6 recipients treated with CD11b^+^Gr1^+^ from donor-derived MDSCs achieved the similar allograft survival with the whole MDSCs treatment group. However, C57BL/6 recipients treated with CD11b^+^Gr1^-^ or CD11b^-^ cells from donor-derived MDSCs did not show significant graft survival benefit (Fig. 6B). Thus, we concluded that the CD11b^+^Gr1^+^ cells, but not non-CD11b^+^Gr1^+^ cells, among donor-derived MDSCs play the key role in the MDSCs mediated allograft survival.

MDSCs are a heterogeneous population of immature monocytes and granulocytes^21^. In addition to Gr-1, CD11b combined with Ly6C and Ly6G has been utilized to distinguish the subpopulations of MDSCs^45–47^: Ly6C^+^Ly6G^-^ (monocytic MDSC, M-MDSC), Ly6G^+^Ly6C^-^ (granulocytic MDSC, G-MDSC) and Ly6C^+^Ly6G^+^ MDSC. Our in vitro generated MDSCs are composed with approximately 47.14% of M-MDSC, 2.74% of G-MDSC and 34.43% of Ly6C^+^Ly6G^+^ MDSC (Fig. S1B). To determine which population plays the key role in the allograft survival prolongation, we flow-sorted the three subsets after MDSC differentiation in vitro and transferred them to recipients 7 days before HTx. Because the dose of whole MDSC treatment group is 1 $\times$ 10^6^, we adjusted the treatment dose to reflect the percentage of specific subset within the whole MDSC, that is: (1) 4.71 $\times$ 10^5^ of M-MDSC; (2) 3.44 $\times$ 10^5^ of Ly6C^+^Ly6G^+^ MDSC; (3) 2.74 $\times$ 10^4^ of G-MDSC. Surprisingly, none of the single population treatment group (M-MDSC MST 7 days, n = 4; Ly6C^+^Ly6G^+^ MDSC MST 7 days, n = 4; G-MDSC MST 7 days n = 3) (Fig. S1C), but only the combination of the 3 populations significant improved allograft survival (whole MDSC MST 14 days, n = 8). This result shows that the 3 different populations of MDSC synergistically suppressed allo-immunity.

### *CD11b*^+^*Gr1*^+^*cells from donor-derived MDSCs, but not from donor-derived cMDCs, suppress alloimmunity*

In vitro generated donor-derived MDSCs contained > 85% CD11b^+^Gr1^+^ cells, while generated donor-derived cMDCs (control) contained only 40% CD11b^+^Gr1^+^ cells (Fig. S1B). To study whether donor MDSCs mediated alloimmune suppression is due to the different dose of CD11b^+^Gr1^+^ cells rather than the different biological properties of cMDCs, we isolated CD11b^+^Gr1^+^ cells from MDSCs and cMDCs by FACS and performed in vitro (Fig. S2A) and in vivo suppression assay (Fig. 6C).

The in vitro study showed that CD11b^+^Gr1^+^ cells from MDSCs suppressed the allo-reactive CD4^+^ as well as CD8^+^ T cell proliferation. In contrast, CD11b^+^Gr1^+^ cells from cMDCs not only failed to suppress the allo-reaction, but also showed enhanced CD8^+^ T cell proliferation (Fig. S2B). In accordance with this finding, C57BL/6 mice treated with CD11b^+^Gr1^+^ cells from cMDCs accelerated the allograft rejection instead of prolongation (n = 4, MST = 6.5 days) (Fig. 6D).

We concluded that the CD11b^+^Gr1^+^ population in MDSCs versus cMDCs carry significantly distinct biological property. Only MDSCs derived CD11b^+^Gr1^+^ cells were able to suppress the alloimmune reaction.

## Discussion

Achieving donor-specific tolerance without compromising the overall immune response is the ultimate goal in transplantation. Regulatory / tolerogenic DC (DCreg/ DCtol) based therapies have been shown to protect allografts and attenuate GvHD^48–50^. Previous study by others have demonstrated that in an NHP allogeneic kidney transplant model, donor-derived DCreg^17^ administration prolonged the allografts survival, which indicated both the safety and efficacy of a single donor-derived DCreg infusion. However, the suppressive function of DCreg was limited due to the maturation and polarization under the stimulation of cytokines secreted by helper T cells during early phase of allo-reaction. MDSCs are diverse collection of immature myeloid-lineage cells, which show overlapping regulatory or suppressive properties with DCregs^51,52^ . As MDSCs show more stable immature biological properties as well as enhanced immune suppressive capabilities^53^, we hypothesized that donor-type MDSCs induce donor-specific immune suppression in solid organ transplantation.

We generated MDSCs from bone marrow cells within 6 days in the presence of GM-CSF^29^, TGFβ^30^ and IL10^31,32^. As IFNγ is essential for the suppression capability of MDSCs via STAT1 signaling activation^54,55^ and iNOS and NO production^56^, we added IFNγ on day 5 to promote the suppressive function of MDSCs^33^ (Fig. S1A). To optimize the MDSCs generation protocol, we compared the biological properties of GM-CSF/IL10/TGFβ1 generated cells, freshly isolated bone marrow cells and myeloid derived cells generated with GM-CSF only (GM-MDCs). Results showed that only MDSCs generated under our protocol had the capability to suppress alloimmune reaction in vitro and in vivo (Figs. S3, S4). As donor-derived MDSCs require 6 days of culture, the clinical application of our approach is more suitable for living donor transplantation, such as lung, kidney and liver. However, we employed in this study a very stringent, reproducible and well established mouse allogeneic cardiac transplantation to test our hypothesis^57^.

Several conclusions are drawn from the current study. First, the in vitro generated MDSCs showed immune suppressive phenotype at the level of both protein and mRNA. Our generated MDSCs not only indicate the ≥ 85% purity of CD11b^+^Gr1^+^ cells but also display a stable immune suppressive phenotype (Fig. S1B). In addition, the low expression of costimulatory molecules, the generated MDSCs display a high level of PD1. Although PD1 is known to be mainly expressed on T cells, evidence has emerged indicating that other non-lymphoid innate cells also express PD1^36^. Recent studies demonstrate that expression of PD1 on myeloid cells reduces proinflammatory cytokine production^58^, diminishes innate immunity against bacterial infection^59^, and suppresses antigen-specific CD8^+^ T cell proliferation via decreasing the production of IL-2 and IFNγ^60^. It is known that successful prevention of acute allorejection using cellular approaches is dependent on migration of suppressor cells to secondary lymphoid organs^61^. Our MDSCs show a high expression of CX3CR1, that not only directs trafficking^62^, but also promotes migration^63,64^. CX3CR1 binds to CX3CL1, a membrane-bound chemokine that is highly expressed in the spleen and lymph nodes, and provides a strong survival signal to MDSCs under both steady-state and inflammatory conditions^65^. Transferred donor CX3CR1^hi^ MDSCs may rapidly migrates into spleen and lymph nodes, thus promoting an immune suppressive microenvironment. We also performed RNA-seq of MDSCs and cMDCs to compare the transcriptomic signatures. When we compared the RNAseq of MDSCs and cMDCs by principal component analysis (PCA), we found that MDSCs phenotypically separated from cMDCs, suggesting distinct transcriptional programs (Fig. S1E). Differential gene expression analysis (Fig. S1D, Table S3) showed the marked up-regulation of Tgfbi in MDSCs. Tgfbi is a secreted protein found in the ECM and it has an N-terminal secretory signal, four FAS1 homologous internal domains, and a cell attachment site (RGD) at its C terminus^66^. Tgfbi binds to the ECM through interaction of the YH motif in its FAS1 domains with collagens I, II, IV, and VI^67,68^. Its FAS1 domain interacts with its α3β1, αVβ3, and αVβ5 integrins on the cell surface. Study has shown that recombinant Tgfbi inhibited the proliferation and activation of CD4^+^ and CD8^+^ T cells stimulated with anti-CD3 mAb via reducing the production of IFNγ and granzyme B in vitro^69^. The mRNA expression of S100A8 and S100A9 is also significantly up-regulated in MDSCs. S100A9 protein plays critical role in inhibition of dendritic cell differentiation and accumulation of MDSCs via up-regulating reactive oxygen species (ROS)^70^. Study of transplant patients has demonstrated that high expression of S100A9 predicts better graft outcomes^71^. In line with these positive clinical outcomes, DCs treated with S100A8 or S100A9 maintain their immature phenotype, and show significant reduction in their capacity to induce T cell proliferation or to produce IFNγ^71^.

We also demonstrated that systemic administration of donor-derived MDSCs induced the endogenous MDSCs, which showed the donor-specific alloimmune suppressive capability. A significant increased population of CD11b^+^Gr1^+^ is observed in donor MDSCs treated recipients’ spleen as well as in allografts. Depletion of this population in vivo abolished the donor MDSCs induced allograft protection. Furthermore, the addition of these recipients’ endogenous MDSCs showed a powerful immune suppression in primary donor type APC (cDCs) stimulated alloMLR system in comparison to the third-party APC stimulated alloreaction, that indicated the immune suppression function of the recipients’ endogenous MDSCs is donor-specific. While the exact mechanism requires more investigation, this observation may be explained in a two-step process. First, pretreatment with donor-derived MDSCs conditions recipients by inhibiting donor-reactive Teff. Pretreatment with donor-type MDSCs acts as an immune suppressive vaccine, which leads to a primary host versus donor antigen response. Recipient’s cognate T cells engage with donor MDSCs presenting allo-antigen with poor co-stimulatory signal leading to the suboptimal activation^72^ which in turn results in the generation of donor- specific Tregs^73^ and anergy of donor-specific Teffs^74^. Our data supports this by showing increase in activated Treg (Fig. S6C) as well as decrease in activated Teff (Fig. 2C). Another important immune reaction take place simultaneously: recipient’s endogenous MDSCs are induced with high level of MHC-II (Fig. S1B) and PDL1 (Fig. 4B) expression. Inhibition of antigen-specific Teff depends on the sufficient level of MHC class II^38^ and PDL1^35,36^ expressed on MDSCs, which was consistent with our Teff analysis results in Figs. 2C and 3. Step 2: After allografts are implanted, the passenger lymphocytes and the graft tissue itself serve as the permanent resource of donor antigens. The microenvironment of the recipients promotes the donor-specific endogenous MDSCs which leads to the donor-specific allograft protection.

In conclusion, this study suggests that systemic administration of donor-derived MDSCs leads to immune regulation in a donor-specific manner via inducing endogenous MDSCs. Further research is needed to determine the detailed mechanism underlying in vivo MDSCs programming and to confirm these findings in human transplant recipients.

## Methods

### Mice

Female mice 6–12 weeks of age were used for all experiments. Wide-type C57BL/6 J (C57BL/6, H2K^b^, I-A^b^), BALB/cByJ (BALB/c, H2K^d^, I-A^d^), C3H/HeJ (C3H, H2K^k^, I-A^k^) mice were from The Jackson Laboratory.

All animal experiments and methods were performed in accordance with the relevant guidelines and regulations approved by the Institutional Animal Care and Use Committee of Brigham and Women’s Hospital, Harvard Medical School, Boston, MA (protocol number: 2016N000242/000,250 2018N000010).

### Generation of MDSCs and cMDCs from bone marrow cells (BMCs)

Femoral and tibial BMCs were obtained from female BALB/c and C3H mice. To generate MDSCs, BMCs were cultured in RPMI-1640 medium (Gibco, 61870036) supplemented with 10% FBS (Gibco, 10082147), 20 ng/mL GM-CSF, IL10 and TGFβ (PeproTech, 315-03, 210-10, 100-21) and 50 mM 2-Mercaptoethanol (Sigma-Aldrich, M6250) for 6 days in 24-well TC-treated plates (Costar, CLS3527). 10 ng/mL IFNγ (PeproTech, 315-05) was added on day 5.

To generate cMDCs, BMCs were cultured in RPMI-1640 supplemented with 10% FCS, 10 ng/mL GM-CSF, IL4 (PeproTech, 214-14) and 50 mM 2-Mercaptoethanol for six days in 24-well cell culture plates. 10 ng/mL IFNγ (PeproTech, 315-05) was added on day 5.

The graphic protocol is shown in Fig. S1A.

### Flow cytometry

Cells were stained with antibodies from Biolegend (anti-CD4, RM4-5; anti-CD8, 53-5.8; anti-CD11b, M1/70; anti-CD11c, N418; anti-Ly6G, 1A8; anti-Ly6C, HK1.4; anti-Gr1, RB6-8C5; anti-MHC-II, M5/114.15.2; anti-CD40, 3/23; anti-CD80, 16-10A1; anti-CD86, GL-1; anti-CX3CR1, SA011F11; anti-PDL1, MIH7; anti-IL4, 11B11; anti-CD44, IM7; anti-CD62L, MEL-14; anti-Ki67, 11F6; anti-CD25, PC61; anti-H2Kb, AF6-88.5; anti-H2Kd, SF1-1.1), eBioscience (anti-FoxP3, FJK-16S). For intracellular staining, FoxP3/Transcription Factor Staining Buffer Set was used (eBioscience, 00-5523-00). Flow cytometry was determined by Canto-II instrument (BD) and analyzed by FlowJo v10.

For cell sorting, CD11b^+^ cells were pre-enriched with anti-CD11b microbeads (Miltenyi Biotec, 130-097-142). Enriched CD11b^+^ cells were stained with anti-CD11b and anti-Gr-1 for FACS by using an MoFlo Astrios system (Beckman Coulter).

Detailed reagents information is listed in Table S2.

### RNA-seq

RNAseq library preparations were performed as previously described^75^. Briefly, samples were lysed with RLT Buffer (Qiagen) and RNA was isolated using MyOne Silane Dynabeads (Thermo Fisher Scientific). RNA was fragmented and barcoded with 8 bp barcodes. Primers were removed using Agencourt AMPure XP bead cleanup (Beckman Coulter/Agencourt). Samples were amplified with 14 PCR cycles. Libraries were gel purified and quantified using a Qubit high sensitivity DNA kit (Invitrogen) and library quality was assessed using Tapestation high sensitivity DNA tapes (Agilent Technologies). RNA was sequenced on an Illumina NextSeq sequencer (Illumina) according to manufacturer’s instructions, sequencing 50 bp single end reads. Analysis was performed using the CLC Genomics Workbench version 8.0.1 RNAseq analysis software package (Qiagen). Briefly, reads were aligned (mismatch cost = 2, insertion cost = 3, deletion cost = 3, length fraction = 0.8, similarity fraction = 0.8) to the mouse genome and differential expression analysis was performed (total count filter cutoff = 5.0). Results were normalized to reads per million.

### Heterotopic cardiac transplantation

All transplant procedures were performed under anesthesia with isoflurane. Fully vascularized heterotopic hearts from BALB/c or C3H were transplanted into C57BL/6 recipients using a microsurgical technique^14,57^. Graft survival was considered complete at the time of cessation of a palpable heart beating and confirmed visually by laparotomy.

### Isolation of lymphocytes from grafts

Tissues were disrupted mechanically in 10 ml digestion solution, which include 0.5 mg/ml collagenase IV (Sigma, 5138), 50 U/ml DNaseI (Invitrogen, 18047019) in RPMI-1640 medium and incubated at 37 °C for 30 min. After that, 10 ml of iced RPMI 1640 with 5% FBS was added. The suspension was filtered through a nylon mesh (70 μm) to remove aggregates. The resulted cell suspension was centrifuged at 800*g* for 5 min to pellet the cells. The pellet was suspended in 5 ml PBS, loaded onto 5 ml of Lympholyte-M (Cedarlane, CL5035) and centrifuged at 1500 g for 25 min at room temperature. Cells were isolated from the Lympholyte-M interface and washed twice in PBS at 300 g for 5 min and prepared for flow cytometry analysis.

### Mixed lymphocyte reaction (MLR)

Allogeneic MLR was performed in duplicate in 96-well, round-bottom plates (Corning, 7007). Nylon wool-eluted spleen T cells (2 × 10^5^/well) were labeled with CellTrace *violet* (Invitrogen, C34557) and used as responders. Cultures were maintained in the complete medium for 3–4 days at 37 °C in 5% CO_2_ in air. The reaction system and other details are shown in the associated figures.

### Histopathology

Grafts harvested on POD7 were fixed in 10% formalin solution (Sigma, HT5011) and then embedded in paraffin. Sections of 4 µm were made for hematoxylin and eosin (H&E) staining.

### Immuno fluorescent staining

Grafts harvested on POD7 were embedded in OCT compound (Sakura, 4583) and preserved in − 80 °C freezer. Cryo sections of 4 µm were made for immuno fluorescent staining^76^. Antibodies used in this experiment is listed in Table S2.

### RNA preparation and quantitative reverse transcriptase-polymerase chain reaction (qRT-PCR)

Cardiac grafts were harvested on POD3 and POD7 and submerged in RNA*later* stabilization for freezing (Sigma, R0901). Total RNA was extracted from frozen tissue samples using TRIzol method (Invitrogen, 15596026). Then, RNA was reverse transcribed to cDNA using iScript cDNA Synthesis Kit (BIO-RAD, 1708891). Quantitative RT-PCR was performed using a SsoAdvance Universal SYBR Green system (BIO-RAD, 1725274) on the QuantStudio 6 Flex Real-Time PCR System (Applied Biosystems). The normalized threshold cycle (Ct) value of each gene was obtained by subtracting the Ct value of 18S rRNA. The sequences used in our study are shown in Table S1.

### MDSCs depletion

Recipients received the intraperitoneal injection of 250 μg RB6-8C5^42–44^ antibody (BioXCell, BE0075) or a rat IgG2β isotype control (BioXCell, BE0090) at POD 0, 2, 4, 6.

### Statistical analysis

The data were analyzed using GraphPad Prism, version 5.0 (GraphPad Software). One-way ANOVA and Tukey's test were used to compare the means of more than two groups. Student's t test was used to compare the means of two groups. A statistical evaluation of graft survival was performed using Kaplan–Meier curves and compared using log-rank tests. RNA-seq data was analyzed by using extraction and analysis of differential gene expression (EDGE) test. All in vitro experimental data were representative of at least three independent experiments. p values of less than 0.05 were considered statistically significant.

## Supplementary information

Supplementary file 1

Supplementary file 2

Supplementary file 3

Supplementary file 4

Supplementary file 5
