# Supplementary material for: Donor myeloid derived suppressor cells (MDSCs) prolong allogeneic cardiac graft survival through programming of recipient myeloid cells in vivo
Source: Sci Rep. 2020 Aug 28;10:14249. doi: 10.1038/s41598-020-71289-z (PMC7455707; doi:10.1038/s41598-020-71289-z)
Supplement: Supplementary file 1 [file 41598_2020_71289_MOESM1_ESM.docx]

Donor Myeloid Derived Suppressor Cells (MDSCs) Prolong Allogeneic Cardiac Graft Survival through programming of Recipient Myeloid Cells *in vivo*

Songjie Cai^1,2,3^, John Y. Choi^1,3^, Thiago J. Borges^1,3^, Hengcheng Zhang^1^, Ji Miao^2^, Takaharu Ichimura^1^, Xiaofei Li^1^, Simiao Xu^2^, Philip Chu^1^, Siawosh K. Eskandari^1^, Hazim Allos^1^, Juliano B. Alhaddad^1^, Saif A. Muhsin^1^, Karim Yatim^1^, Leonardo V. Riella^1^, Peter T. Sage^1^, Anil K. Chandraker^1^, Jamil R. Azzi ^1^

^1^ Transplantation Research Center, Renal Division, Brigham and Women's Hospital, Harvard Medical School, Boston, MA.

^2^ Division of Endocrinology, Boston Children's Hospital, Harvard Medical School, Boston, MA.

^3^ These authors contributed equally to this work.

**Supplementary figures with figure legends**


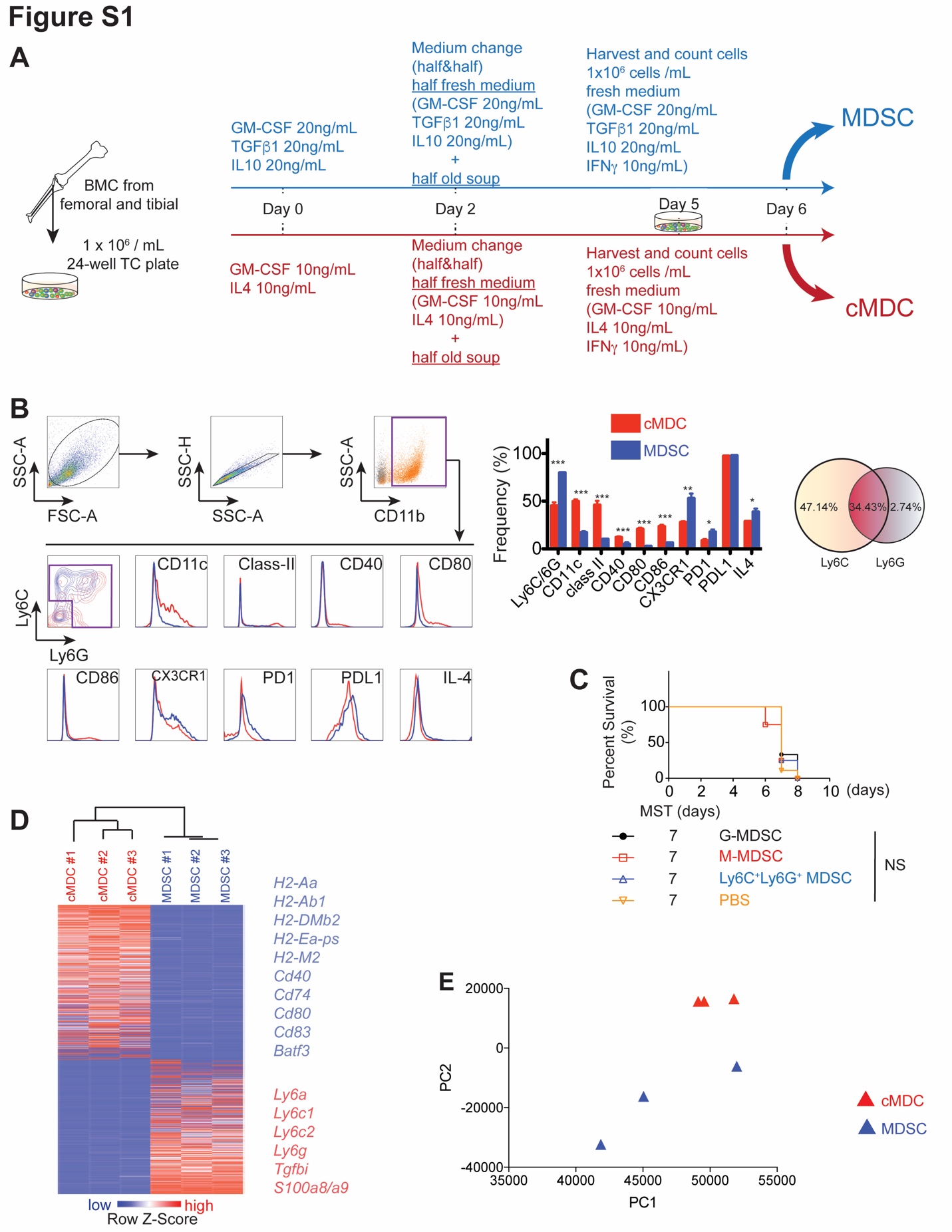


**Figure S1.** **(A)** This schematic model shows the culture protocol of MDSCs as well as control cMDCs. **(B)** Flow cytometry analysis of MDSCs and control cMDCs (n=4 per group). Protein expression represented as frequency. Mean ± SEM, * p<0.05, ** p< 0.01, *** p<0.001, two-tailed unpaired t test. Data represents one of 4 separate experiments. **(C)** The allograft survival of C57BL/6 recipients received the three subsets of BALB/c donor-type derived MDSCs treatment. The treatment dose of each group consistent with the approximate percentage of the whole MDSC, which is: 1)- 4.71$\times$10^5^ of Ly6C^+^Ly6G^-^ M-MDSC (n=4); 2)- 3.44$\times$10^5^ of Ly6C^+^Ly6G^+^ MDSC (n=4); 3)- 2.74$\times$10^4^ of Ly6C^+^Ly6G^-^ G-MDSC (n=3). Kaplan-Meier cumulative survival of allograft show none of the single population treatment did prolong the allografts survival (log-rank test). **(D)** Heatmap displaying normalized expression of top 182 significant genes in MDSCs and cMDCs (n=3 per group). The MDSCs suppressive function related genes were lined up. p<0.05, EDGE test. **(E)** Principal component analysis of RNAseq of MDSCs and cMDCs.


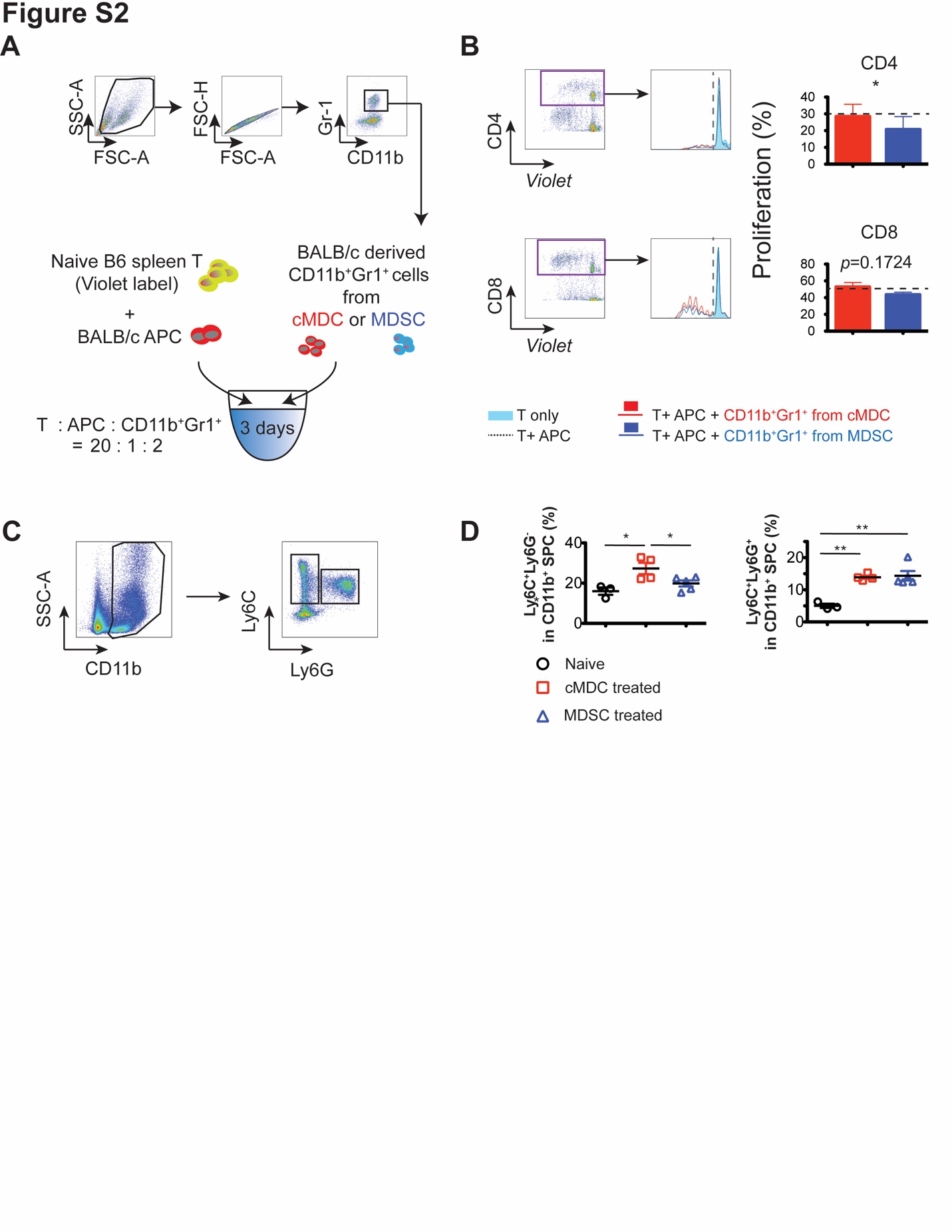


**Figure S2.** **(A)** Schematic diagram of the experimental design of the study. **(B)** CD4^+^ and CD8^+^ T cell proliferation in response to BALB/c APC was analyzed by CellTrace Violet dye dilution (n=3 per group). Graphs showed the proliferation rate of CD4^+^ and CD8^+^ T cells in the presence of CD11b^+^Gr1^+^ cells from MDSC compared to CD11b^+^Gr1^+^ cells from cMDC. The black dash line indicated the proliferation rate of the reaction system (T + APC). Mean ± SEM, * p<0.05, two-tailed unpaired t test. Data represents one of 3 separate experiments. **(C-D)** Splenocytes were harvested at POD7 for flow cytometry assay. Data indicated that the induced endogenous MDSC were composed of M-MDSC and Ly6C^+^Ly6G^+^ MDSC. There was no difference of Ly6C^+^Ly6G^+^ MDSC population between cMDC and MDSC treated recipients. Mean ± SEM, * p<0.05, ** p< 0.01, one-way ANOVA and Tukey’s test. Data represents one of 3 separate experiments.


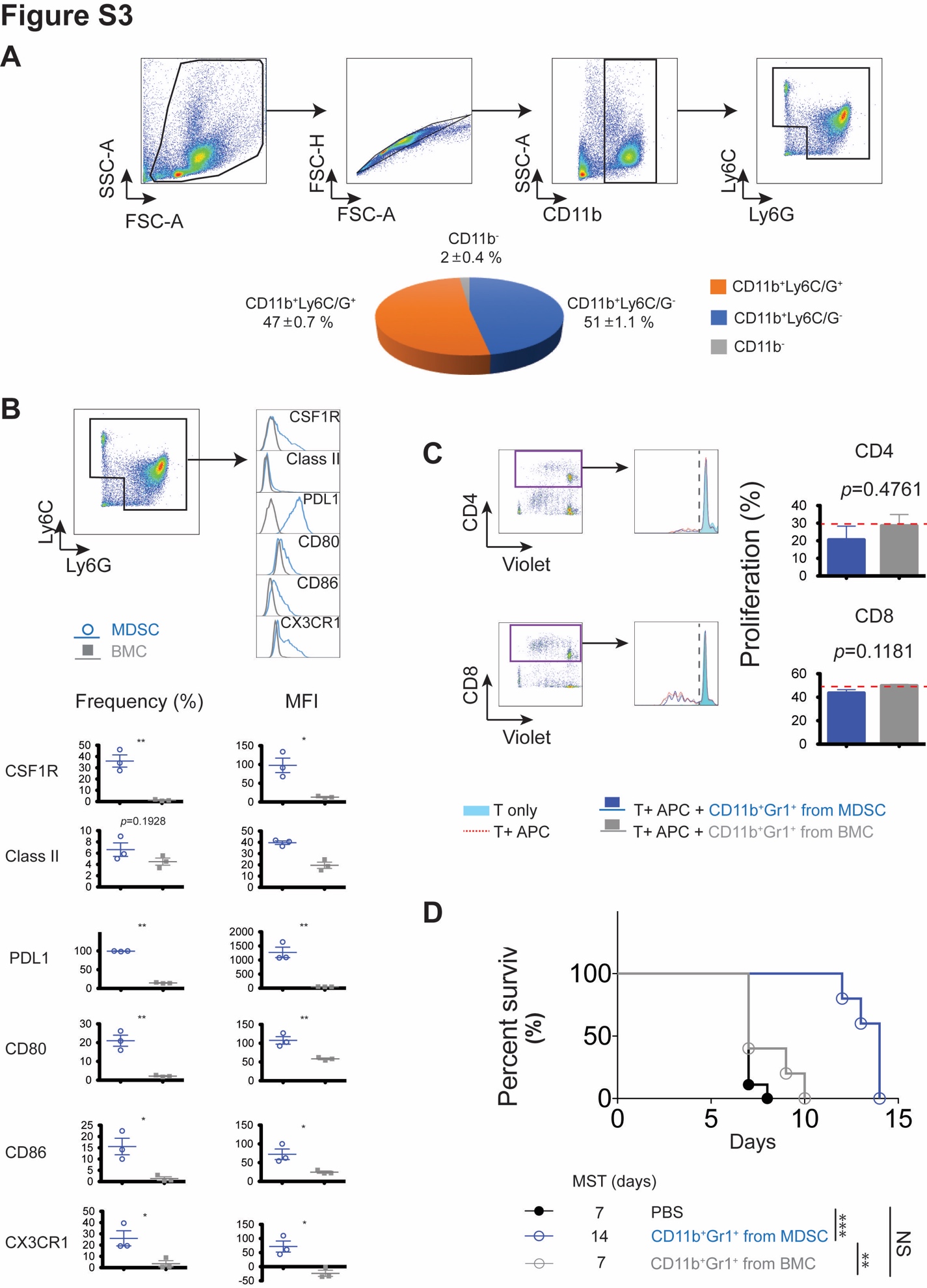


**Figure S3.** **(A)** Flow cytometry analysis showed approximately 47% CD11b^+^ Ly6C/G^+^ cells naturally exist in fresh BMC. **(B)** Gating on CD11b^+^ Ly6C/G^+^, protein expression represented as frequency (left) and MFI (right). Mean ± SEM, * p<0.05, ** p<0.01, two-tailed unpaired t test. Data represents one of 3 separate experiments. **(C)** CD4^+^ and CD8^+^ T cell proliferation in response to allogeneic APC was analyzed by CellTrace Violet dye dilution (n=3 per group). Graphs showed the proliferation rate of CD4^+^ and CD8^+^ T cells in the presence of CD11b^+^Gr1^+^ cells from fresh BMC compared to CD11b^+^Gr1^+^ cells from MDSC. The red dash line indicated the proliferation rate of the reaction system (T + APC). Mean ± SEM, * p<0.05, two-tailed unpaired t test. Data represents one of 3 separate experiments. (**D**) C57BL/6 recipients received single-dose intravenous injection of 1$\times$10^6^ CD11b^+^Gr1^+^ cells from BALB/c BMC or MDSC 7 days prior to cardiac transplantation. Kaplan-Meier cumulative survival of allograft showed CD11b^+^Gr1^+^ cells from BMC failed to prolong allograft survival. ** p< 0.01, *** p<0.001, log-rank test.


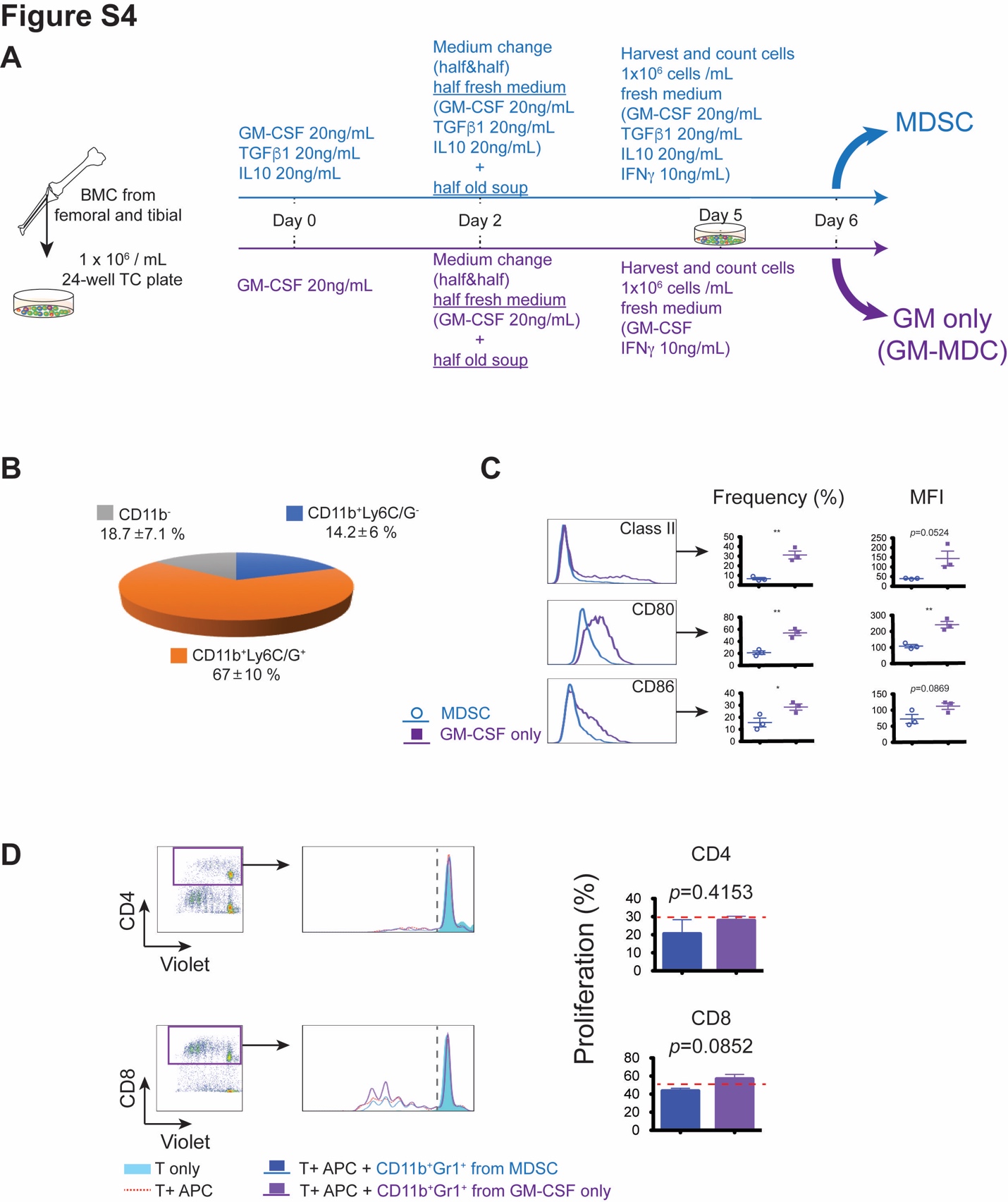


**Figure S4.** **(A)** This schematic model shows the culture protocol of GM-only MDCs. **(B)** Flow cytometry analysis showed approximately 67% CD11b^+^ Ly6C/G^+^ cells in GM-CSF generated cells. **(C)** Gating on CD11b^+^ Ly6C/G^+^, protein expression represented as frequency (left) and MFI (right). Mean ± SEM, * p<0.05, ** p<0.01, two-tailed unpaired t test. Data represents one of 3 separate experiments. (**D**) CD4^+^ and CD8^+^ T cell proliferation in response to allogeneic APCs was analyzed by CellTrace Violet dye dilution (n=3 per group). Graphs showing the proliferation rate of CD4^+^ and CD8^+^ T cells in the presence of CD11b^+^Gr1^+^ cells from GM-CSF generated cells *vs.* CD11b^+^Gr1^+^ cells from MDSCs. The red dash line indicates the proliferation rate of the reaction system (T + APC). Mean ± SEM, two-tailed unpaired t test. Data represents one of 3 separate experiments.


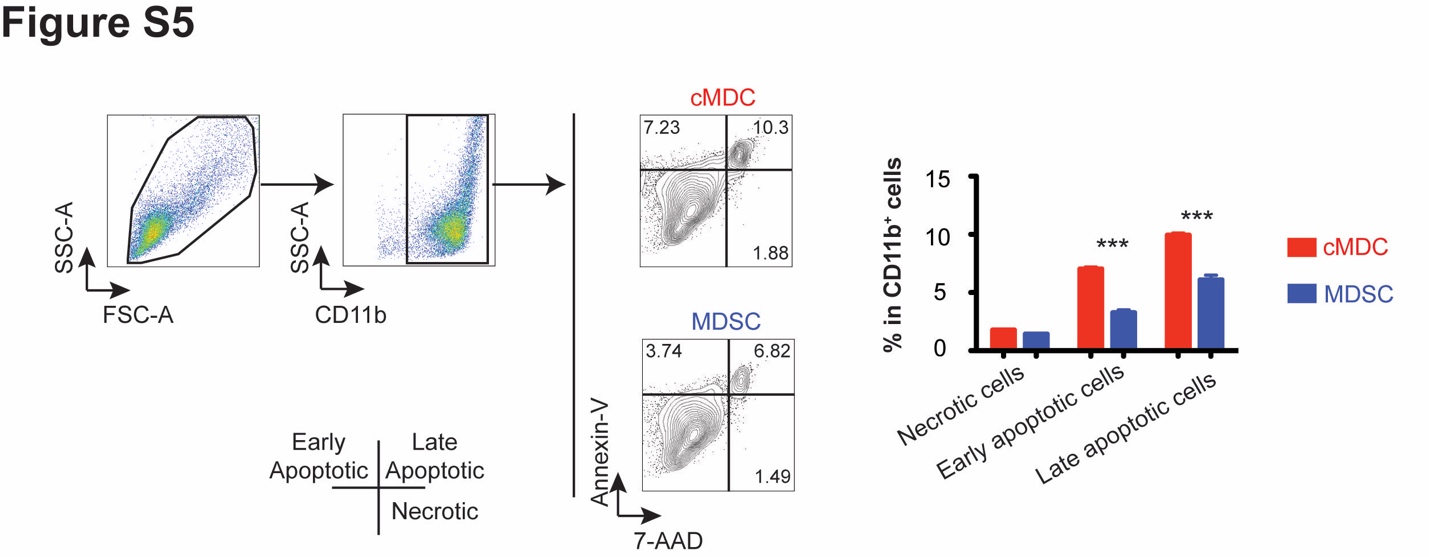


**Figure S5.** *In vitro* generated MDSCs and the control cMDCs were stained with APC/Cy7 CD11b, Pacific Blue Annexin-V and 7-AAD. Gating on CD11b^+^ cells, the necrotic and apoptotic cells frequency was observed by measuring the percentage of Annexin-V and 7-AAD negative and positive cells. Mean ± SEM, *** p<0.001, two-tailed unpaired t test. Data represents one of 3 separate experiments.


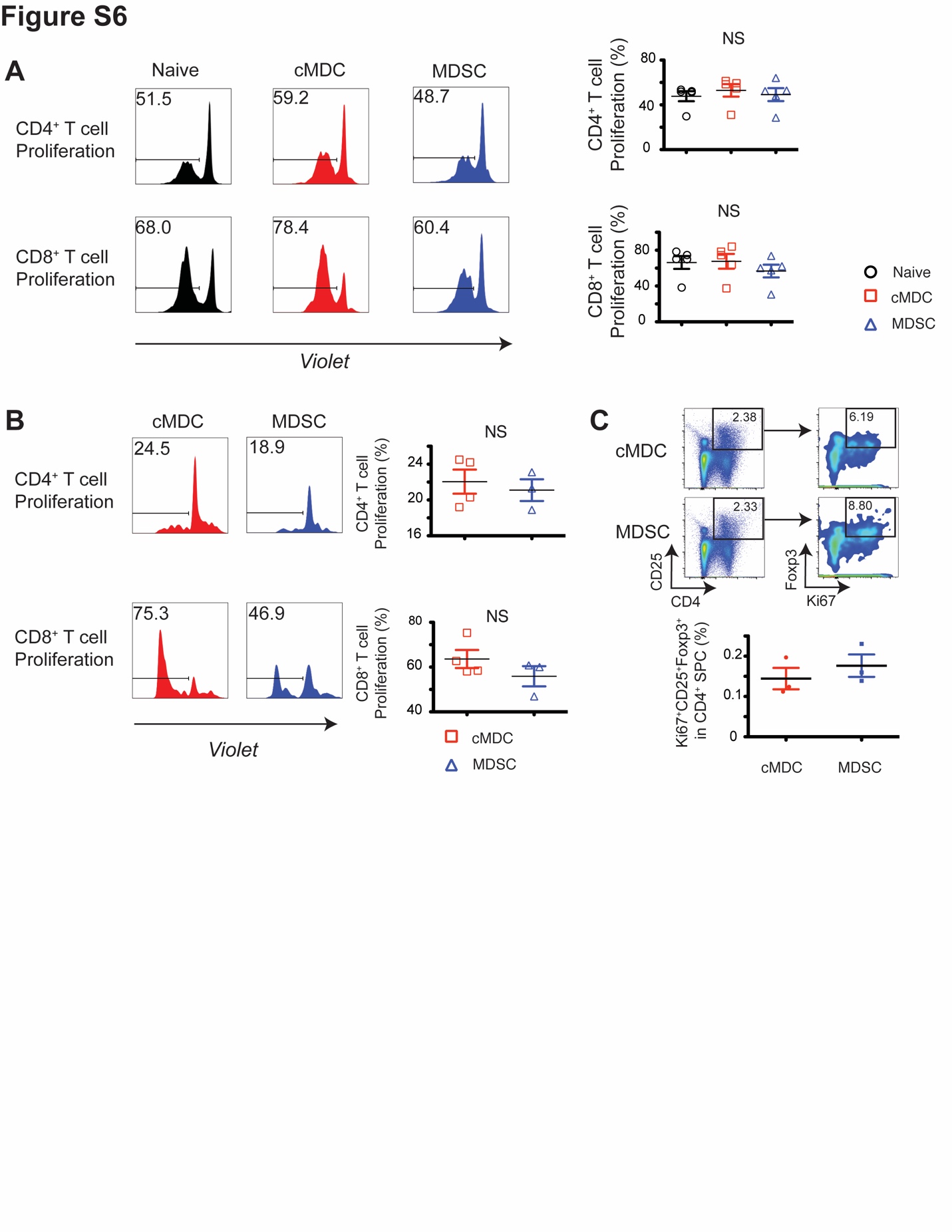


**Figure S6**: **(A)** T cell proliferation assay using Violet CellTrace co-stained with CD4 and CD8, which relates to **Figure 3B** in manuscript. Mean ± SEM, one-way ANOVA and Tukey’s test. **(B)** T cell proliferation assay using Violet CellTrace co-stained with CD4 and CD8, which relates to **Figure 4B** in manuscript. Mean ± SEM, two-tailed unpaired t test. **(C)** Recipients were taken down on POD7. Splenocytes were stained with CD4, CD25, FoxP3 and Ki67 for activated Treg assay. The frequency of activated Tregs (CD25^+^FoxP3^+^Ki67^+^) in CD4^+^ solenocytes increased in MDSC treated recipients compared to cMDC group. Mean ± SEM, two-tailed unpaired t test.


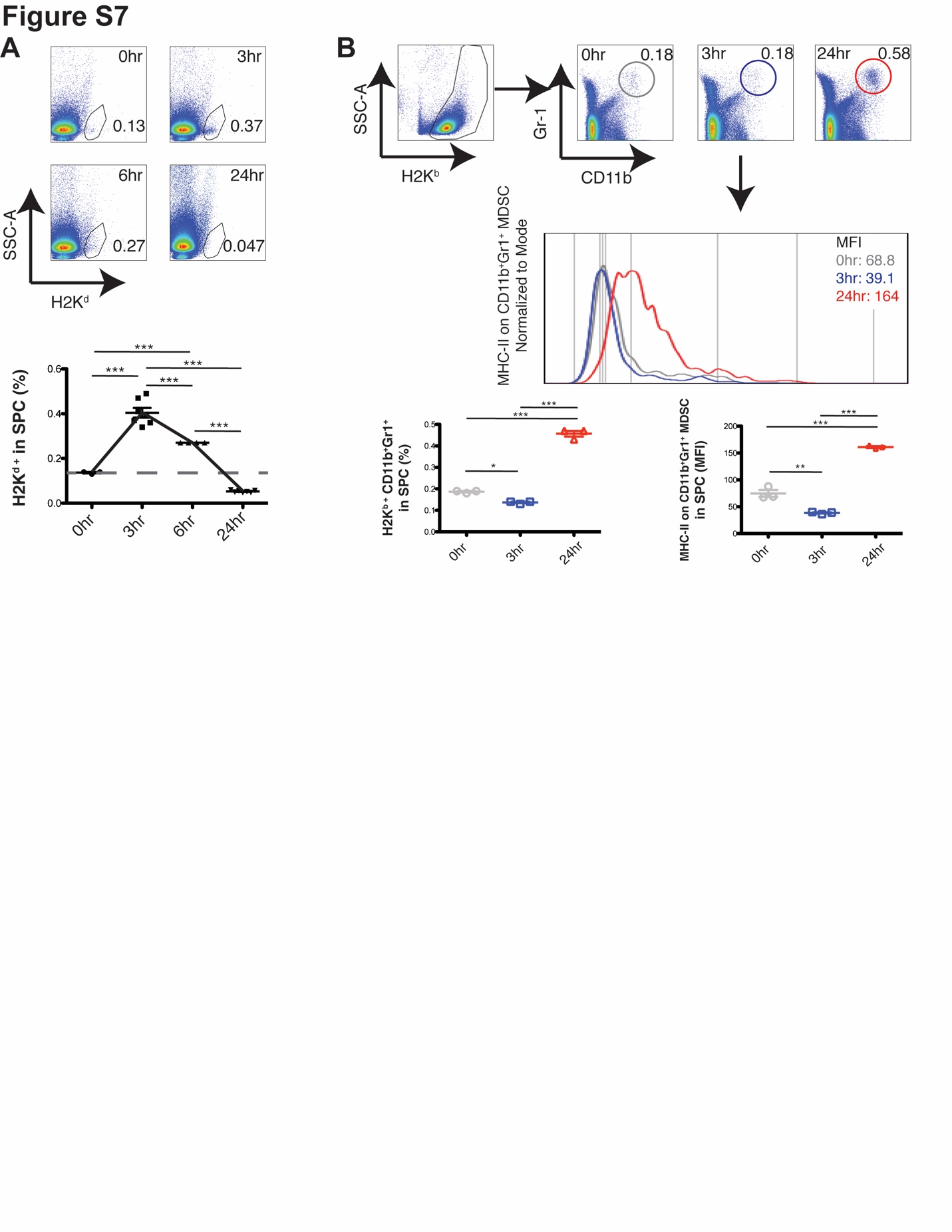


**Figure S7**: 1$\times$10^6^ BALB/c MDSCs were intravenously injected to C57BL/6 recipients. Recipients were sacrificed at 3, 6, 24 hours post injection for flow cytometry. **(A)** Splenocytes from recipients were stained with H2K^d^ to trace the infused MDSCs. **(B)** Splenocytes from recipients were stained with H2K^b^, CD11b, Gr-1 and IA/IE to detect the donor derived MDSCs. Mean ± SEM, * p<0.05, ** p<0.01, *** p<0.001, one-way ANOVA and Tukey’s test.


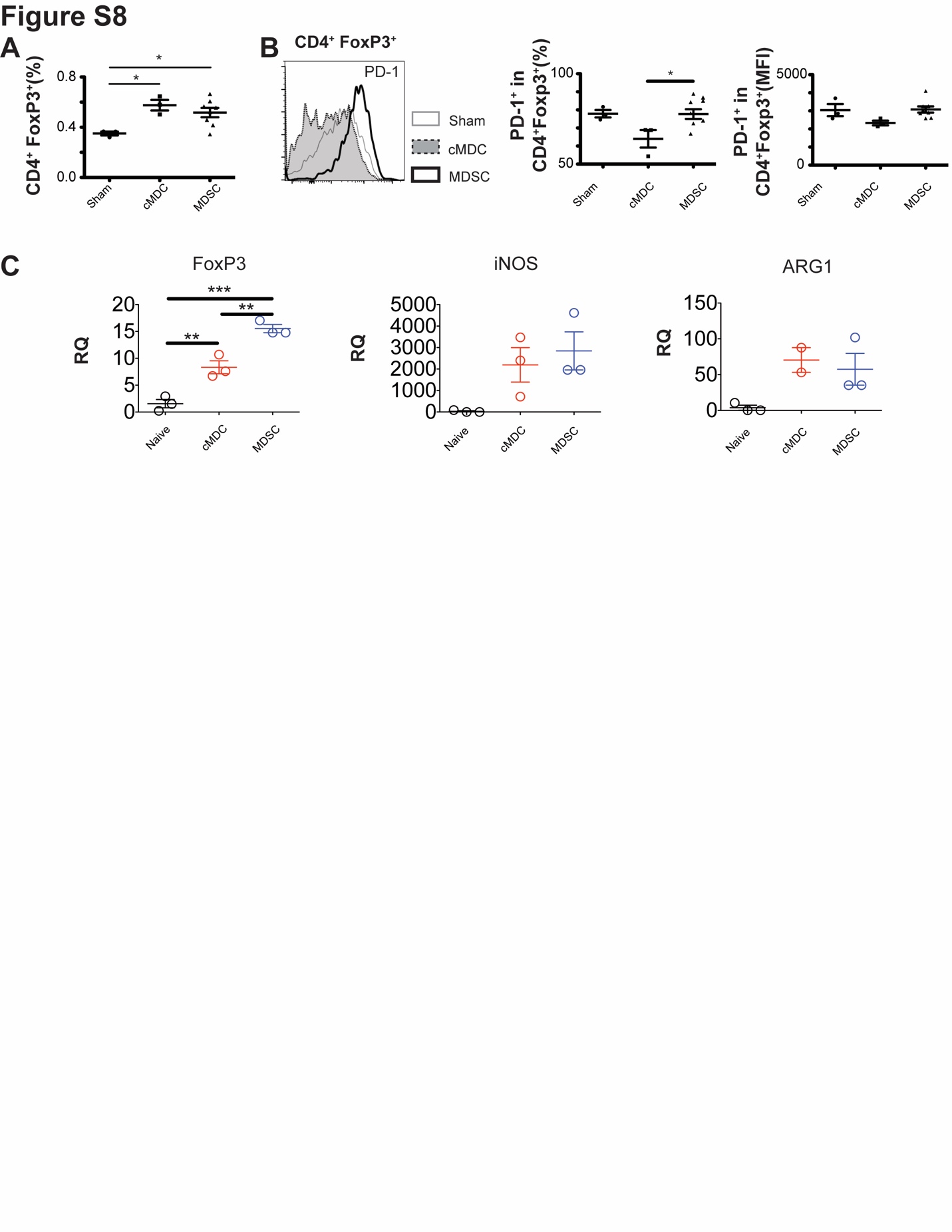


**Figure S8: (A)** 1$\times$10^6^ BALB/c MDSCs were intravenously injected to C57BL/6 recipients. Recipients were sacrificed 7 days post injection for flow cytometry. Splenocytes from recipients were stained with CD4, FoxP3 and PD1. **(B)** Allografts were harvested at POD3 for qRT-PCR. qRT-PCR analysis of allografts (whole tissues) at POD3. Graph represented as RQ (relative quantification) = 2^-ΔΔCt^. Mean ± SEM, ** p< 0.01, ***p<0.001, one-way ANOVA and Tukey’s test.
