# Supplementary material for: Donor myeloid derived suppressor cells (MDSCs) prolong allogeneic cardiac graft survival through programming of recipient myeloid cells in vivo
Source: Sci Rep. 2020 Aug 28;10:14249. doi: 10.1038/s41598-020-71289-z (PMC7455707; doi:10.1038/s41598-020-71289-z)
Supplement: Supplementary file 2 [file 41598_2020_71289_MOESM2_ESM.docx]

Donor Myeloid Derived Suppressor Cells (MDSCs) Prolong Allogeneic Cardiac Graft Survival through programming of Recipient Myeloid Cells *in vivo*

Songjie Cai^1,2,3^, John Y. Choi^1,3^, Thiago J. Borges^1,3^, Hengcheng Zhang^1^, Ji Miao^2^, Takaharu Ichimura^1^, Xiaofei Li^1^, Simiao Xu^2^, Philip Chu^1^, Siawosh K. Eskandari^1^, Hazim Allos^1^, Juliano B. Alhaddad^1^, Saif A. Muhsin^1^, Karim Yatim^1^, Leonardo V. Riella^1^, Peter T. Sage^1^, Anil K. Chandraker^1^, Jamil R. Azzi ^1^

^1^ Transplantation Research Center, Renal Division, Brigham and Women's Hospital, Harvard Medical School, Boston, MA.

^2^ Division of Endocrinology, Boston Children's Hospital, Harvard Medical School, Boston, MA.

^3^ These authors contributed equally to this work.

**Table S1. Primers used in qRT-PCR.**

| *Gene* | Forward Primer (5' -> 3') | Reverse Primer (5' -> 3') |
| --- | --- | --- |
| Rn18S | GCAATTATTCCCCATGAACG | GGCCTCACTAAACCATCCAA |
| Il1a | GCACCTTACACCTACCAGAGT | AAACTTCTGCCTGACGAGCTT |
| Il1b | CCTTCCAGGATGAGGACATGA | TGAGTCACAGAGGATGGGCTC |
| Il2 | TGAGCAGGATGGAGAATTACAGG | GTCCAAGTTCATCTTCTAGGCAC |
| Il4 | AACGAGGTCACAGGAGAAGG | TCTGCAGCTCCATGAGAACA |
| Il10 | GCTCTTACTGACTGGCATGAG | CGCAGCTCTAGGAGCATGTG |
| Il13 | GCAGCATGGTATGGAGTGTG | TGGCGAAACAGTTGCTTTGT |
| Nlrp3 | ATTACCCGCCCGAGAAAGG | TCGCAGCAAAGATCCACACAG |
| Nfe2l2 | TCTTGGAGTAAGTCGAGAAGTGT | GTTGAAACTGAGCGAAAAAGGC |
| Ifng | TTGAGGTCAACAACCCACAG | TCAGCAGCGACTCCTTTTC |
| Irf1 | ATGCCAATCACTCGAATGCG | TTGTATCGGCCTGTGTGAATG |
| Tnf | ATGAGAAGTTCCCAAATGGC | CTCCACTTGGTGGTTTGCTA |
| Tgfb1 | CAACAATTCCTGGCGTTACCTTGG | GAAAGCCCTGTATTCCGTCTCCTT |
| Gzmb | CCACTCTCGACCCTACATGG | GGCCCCCAAAGTGACATTTATT |
| Prf1 | AGCACAAGTTCGTGCCAGG | GCGTCTCTCATTAGGGAGTTTTT |
| Stat5a | CGCCAGATGCAAGTGTTGTAT | TCCTGGGGATTATCCAAGTCAAT |
| Stat5b | CGATGCCCTTCACCAGATG | AGCTGGGTGGCCTTAATGTTC |
| Ly6c | GCAGTGCTACGAGTGCTATGG | ACTGACGGGTCTTTAGTTTCCTT |
| Cd274 | GCTCCAAAGGACTTGTACGTG | TGATCTGAAGGGCAGCATTTC |
| Csf1r | TGTCATCGAGCCTAGTGGC | CGGGAGATTCAGGGTCCAAG |
| Hif1a | GCGAGAACGAGAAGAAAAAGATGA | GGGGAAGTGGCAACTGATGA |
| Foxp3 | GGCCCTTCTCCAGGACAGA | GCTGATCATGGCTGGGTTGT |
