# Supplementary material for: Donor myeloid derived suppressor cells (MDSCs) prolong allogeneic cardiac graft survival through programming of recipient myeloid cells in vivo
Source: Sci Rep. 2020 Aug 28;10:14249. doi: 10.1038/s41598-020-71289-z (PMC7455707; doi:10.1038/s41598-020-71289-z)
Supplement: Supplementary file 3 [file 41598_2020_71289_MOESM3_ESM.docx]

Donor Myeloid Derived Suppressor Cells (MDSCs) Prolong Allogeneic Cardiac Graft Survival through programming of Recipient Myeloid Cells *in vivo*

Songjie Cai^1,2,3^, John Y. Choi^1,3^, Thiago J. Borges^1,3^, Hengcheng Zhang^1^, Ji Miao^2^, Takaharu Ichimura^1^, Xiaofei Li^1^, Simiao Xu^2^, Philip Chu^1^, Siawosh K. Eskandari^1^, Hazim Allos^1^, Juliano B. Alhaddad^1^, Saif A. Muhsin^1^, Karim Yatim^1^, Leonardo V. Riella^1^, Peter T. Sage^1^, Anil K. Chandraker^1^, Jamil R. Azzi ^1^

^1^ Transplantation Research Center, Renal Division, Brigham and Women's Hospital, Harvard Medical School, Boston, MA.

^2^ Division of Endocrinology, Boston Children's Hospital, Harvard Medical School, Boston, MA.

^3^ These authors contributed equally to this work.

**Table S2.** Antibodies used in Flowcytometry analysis and Immune fluorescent staining.

| **ANTIBODY** | **SOURCE** | **CLONE** | **IDENTIFIER (Cat.)** | **APPLICATION SHOWN IN FIGURE** |
| --- | --- | --- | --- | --- |
| **Flowcytometry** | | | | |
| PE/Cy7 anti-mouse CD4 Antibody | BioLegend | RM4-5 | 100528 | Fig 1B, 2C, 3B, 4D, 5D, S2B, S3C, S4D |
| APC anti-mouse CD8a Antibody | BioLegend | 53-6.7 | 100712 | Fig 1B, 2C, 3B, 5D, S2B, S4D |
| Brilliant Violet 421 anti-mouse FOXP3 Antibody | BioLegend | MF-14 | 126419 | Fig 2C, 3B |
| APC-eFluor780 anti h/m CD44 Monoclonal Antibody | eBioscience | IM7 | 47-0441-82 | Fig 2C, 3B |
| APC anti-mouse CD62L Antibody | BioLegend | MEL-14 | 104412 | Fig 2C, 3B |
| Alexa Fluor 488 anti-mouse Ki-67 Antibody | BioLegend | 16A8 | 652418 | Fig 2C, 3B, 4D |
| Brilliant Violet 510 anti-mouse CD8a Antibody | BioLegend | 53-6.7 | 100752 | Fig 2C, 3B |
| APC/Cy7 anti-mouse/human CD11b Antibody | BioLegend | M1/70 | 101226 | Fig 4A, 5C, 6A, 6C, 6E, S1B, S2A, S3A, S4C, S9B |
| PerCP/Cyanine5.5 anti-mouse Ly-6G/Ly-6C (Gr-1) Antibody | BioLegend | RB6-8C5 | 108428 | Fig 4A, 9B |
| PE/Cy7 anti-mouse CD274 (B7-H1, PD-L1) Antibody | BioLegend | 10F.9G2 | 124314 | Fig 4B, S1B, S3B |
| FOXP3 Monoclonal Antibody (FJK-16s), APC, eBioscience | eBioscience | FJK-16s | 17-5773-82 | Fig 4D |
| FITC anti-mouse Ly-6G/Ly-6C (Gr-1) Antibody | BioLegend | RB6-8C5 | 108406 | Fig 5C, 6A, 6C, S2A |
| 7-AAD | Invitrogen |  | A1310 | Fig 6E |
| Pacific Blue Annexin V | BioLegend |  | 640918 | Fig 6E |
| Brilliant Violet 510 anti-mouse CD11c Antibody | BioLegend | N418 | 117353 | Fig S1B |
| PerCP/Cy5.5 anti-mouse Ly-6G Antibody | BioLegend | 1A8 | 127616 | Fig S1B, S3A, S3B, S4B, S4C |
| Brilliant Violet 421 anti-mouse Ly-6G/Ly-6C (Gr-1) Antibody | BioLegend | RB6-8C5 | 108445 | Fig S1B, S3A, S3B, S4B, S4C |
| PE anti-mouse I-A/I-E Antibody | BioLegend | M5/114.15.2 | 107608 | Fig S1B |
| FITC anti-mouse CD279 (PD-1) Antibody | BioLegend | 29F.1A12 | 135214 | Fig S1B |
| PE anti-mouse CD40 Antibody | BioLegend | 3/23 | 124610 | Fig S1B, S3B |
| Brilliant Violet 421 anti-mouse CD80 Antibody | BioLegend | 16-10A1 | 104726 | Fig S1B, S3B, S4C |
| PE/Cy7 anti-mouse CD86 Antibody | BioLegend | GL-1 | 105014 | Fig S1B, S3B, S4C |
| Brilliant Violet 510 anti-mouse CX3CR1 Antibody | BioLegend | SA011F11 | 149025 | Fig S1B, S3B |
| APC anti-mouse IL-4 Antibody | BioLegend | 11B11 | 504106 | Fig S1B |
| Brilliant Violet 421 anti-mouse CD115 (CSF-1R) Antibody | BioLegend | AFS98 | 135513 | Fig S3B |
| FITC anti H2Kb | BioLegend | AF6-88.5 | 116505 | Fig S9B |
| APC anti mouse H2Kd | BioLegend | SF1-1.1 | 116619 | Fig S9A |
| PE anti mouse I-A/I-E | BioLegend | M5/114.15.2 | 107607 | Fig S9B |
| FITC anti-mouse I-A/I-E Antibody | BioLegend | M5/114.15.2 | 107606 | Fig S3B, S4C |
|  |  |  |  |  |
| **IHC** | | | | |
| Purified anti-mouse CD3ε Antibody | BioLegend | 145-2C11 | 100302 | Fig 2B |
| Purified anti-mouse/human CD11b Antibody | BioLegend | M1/70 | 101202 |  |
| Goat anti-Rat IgM (Heavy chain) Cross-Adsorbed Secondary Antibody, Alexa Fluor 488 | Invitrogen |  | A-21212 |  |
