# Supplementary material for: Donor myeloid derived suppressor cells (MDSCs) prolong allogeneic cardiac graft survival through programming of recipient myeloid cells in vivo
Source: Sci Rep. 2020 Aug 28;10:14249. doi: 10.1038/s41598-020-71289-z (PMC7455707; doi:10.1038/s41598-020-71289-z)
Supplement: Supplementary file 4 [file 41598_2020_71289_MOESM4_ESM.docx]

Donor Myeloid Derived Suppressor Cells (MDSCs) Prolong Allogeneic Cardiac Graft Survival through programming of Recipient Myeloid Cells *in vivo*

Songjie Cai^1,2,3^, John Y. Choi^1,3^, Thiago J. Borges^1,3^, Hengcheng Zhang^1^, Ji Miao^2^, Takaharu Ichimura^1^, Xiaofei Li^1^, Simiao Xu^2^, Philip Chu^1^, Siawosh K. Eskandari^1^, Hazim Allos^1^, Juliano B. Alhaddad^1^, Saif A. Muhsin^1^, Karim Yatim^1^, Leonardo V. Riella^1^, Peter T. Sage^1^, Anil K. Chandraker^1^, Jamil R. Azzi ^1^

^1^ Transplantation Research Center, Renal Division, Brigham and Women's Hospital, Harvard Medical School, Boston, MA.

^2^ Division of Endocrinology, Boston Children's Hospital, Harvard Medical School, Boston, MA.

^3^ These authors contributed equally to this work.

**Table S3. Fold changes and P-value of the signature genes obtained from RNA-seq of MDSCs vs. cMDCs.**

***Down regulated genes***

| *Gene* | log2 fold change | P-value |
| --- | --- | --- |
| Ccr7 | -4793.220499 | 5.87E-14 |
| Ccl22 | -446.2020235 | 9.05E-13 |
| Ccl17 | -385.6061393 | 2.75E-12 |
| Cd36 | -324.4358332 | 5.34E-12 |
| Aldh1a2 | -2786.877408 | 1.08E-11 |
| Mgl2 | -105.402338 | 3.36E-10 |
| Retnla | -304.8059888 | 3.87E-10 |
| Flt1 | -155.2180495 | 2.16E-09 |
| Nr4a3 | -239.2867511 | 4.39E-09 |
| Traf1 | -1622.406592 | 1.14E-08 |
| Batf3 | -248.2205664 | 1.49E-08 |
| Slc30a4 | -1313.515011 | 3.03E-08 |
| Lpl | -31.36864558 | 5.40E-08 |
| Ramp3 | -340.8653073 | 9.42E-08 |
| Syt7 | -1049.867067 | 1.12E-07 |
| Fscn1 | -37.14514797 | 1.56E-07 |
| Flrt2 | -330.763887 | 1.96E-07 |
| Hebp2 | -942.1216428 | 3.35E-07 |
| Ear11 | -143.7518343 | 1.10E-06 |
| Serpinb9 | -62.35850379 | 1.23E-06 |
| Ckb | -230.5927308 | 1.57E-06 |
| Ccl24 | -33.2871445 | 1.67E-06 |
| Matk | -702.2861211 | 2.31E-06 |
| Asgr2 | -758.8549786 | 2.79E-06 |
| Palld | -68.41485397 | 3.45E-06 |
| Ocstamp | -683.7791866 | 3.56E-06 |
| Mmp12 | -17.21086831 | 4.60E-06 |
| Apol7c | -587.2364387 | 5.07E-06 |
| AY761185 | -564.0064112 | 5.37E-06 |
| Ids | -125.6108311 | 5.48E-06 |
| Il7r | -120.4227574 | 5.54E-06 |
| P2rx5 | -171.022032 | 5.89E-06 |
| Gm15056 | -175.2411013 | 5.98E-06 |
| Stk39 | -576.5035385 | 6.33E-06 |
| Pvrl2 | -110.1017888 | 6.81E-06 |
| Arg1 | -15.93832847 | 7.78E-06 |
| Cd200 | -553.9665866 | 1.05E-05 |
| Mras | -514.9089221 | 1.14E-05 |
| Fam115c | -167.0526033 | 1.37E-05 |
| Cacna1d | -501.0591701 | 1.51E-05 |
| Fyn | -119.6852651 | 1.67E-05 |
| Vdr | -75.39609783 | 1.73E-05 |
| Kcp | -156.3033753 | 1.77E-05 |
| Slamf8 | -99.55243931 | 2.22E-05 |
| Sdc1 | -31.13835637 | 2.36E-05 |
| Emp2 | -458.0036895 | 2.51E-05 |
| Ccl5 | -36.94300756 | 2.57E-05 |
| Asb2 | -539.6921465 | 2.59E-05 |
| Gimap4 | -414.1946599 | 2.72E-05 |
| Lad1 | -488.9553659 | 2.79E-05 |
| Cxcl9 | -642.1934927 | 4.33E-05 |
| Tnfsf15 | -386.7086817 | 4.63E-05 |
| Tarbp2 | -63.42758113 | 5.04E-05 |
| Rhebl1 | -377.7241227 | 5.44E-05 |
| Gpr65 | -446.7668092 | 5.71E-05 |
| Dusp22 | -391.4034604 | 5.95E-05 |
| Crip1 | -25.24354098 | 6.99E-05 |
| Abcg2 | -104.6897967 | 8.18E-05 |
| Dpy19l3 | -69.28640371 | 8.50E-05 |
| Tnip3 | -45.15500038 | 9.00E-05 |
| Stk11ip | -98.40418819 | 9.41E-05 |
| Clybl | -327.8923814 | 1.06E-04 |
| Gm17511 | -399.0139048 | 1.10E-04 |
| Nedd4l | -73.92650161 | 1.21E-04 |
| Man1a | -314.9657553 | 1.21E-04 |
| Polrmt | -343.7257077 | 1.27E-04 |
| Klhl22 | -307.2397679 | 1.30E-04 |
| Thop1 | -325.4597007 | 1.54E-04 |
| Ubd | -120.5206559 | 1.59E-04 |
| Lmo1 | -89.94793396 | 1.65E-04 |
| Arap2 | -86.65324951 | 1.70E-04 |
| Fabp5 | -11.81549932 | 1.98E-04 |
| Phf13 | -281.9648314 | 2.00E-04 |
| Irf4 | -10.53523883 | 2.01E-04 |
| Pkib | -11.80758257 | 2.02E-04 |
| Ppp2r3a | -63.63462438 | 2.04E-04 |
| Lbx2 | -277.851856 | 2.12E-04 |
| Slamf7 | -334.272179 | 2.22E-04 |
| Hemk1 | -13.92647763 | 2.39E-04 |
| Mgarp | -783.3248462 | 2.44E-04 |
| Lef1 | -346.7414142 | 2.69E-04 |
| Wdr91 | -285.3689801 | 2.82E-04 |
| Tmsb10 | -8.95308649 | 2.89E-04 |
| Il33 | -564.6252966 | 2.92E-04 |
| Sema7a | -260.7385635 | 3.06E-04 |
| Tnfrsf9 | -51.63854014 | 3.10E-04 |
| Sdc3 | -23.14901928 | 3.18E-04 |
| Slc7a2 | -9.177468705 | 3.22E-04 |
| Snx8 | -36.10847395 | 3.22E-04 |
| Adam23 | -369.0721227 | 3.30E-04 |
| Gpt2 | -350.7671335 | 3.40E-04 |
| Cd74 | -7.6773841 | 3.45E-04 |
| Arhgap6 | -65.36984653 | 3.46E-04 |
| Cd83 | -34.38412826 | 3.53E-04 |
| Rab39 | -294.4815246 | 3.77E-04 |
| Odf2l | -299.735257 | 3.79E-04 |
| S100a4 | -14.35002683 | 3.80E-04 |
| Tmem237 | -324.9106358 | 3.88E-04 |
| Tmem132a | -56.9489486 | 3.95E-04 |
| Anxa4 | -7.894051865 | 4.00E-04 |
| Ankrd66 | -273.7369302 | 4.02E-04 |
| Stac2 | -234.4641418 | 4.03E-04 |
| Acss1 | -245.6173842 | 4.13E-04 |
| Lgals4 | -248.7903752 | 4.32E-04 |
| Vopp1 | -29.36958057 | 4.38E-04 |
| 2310047M10Rik | -56.98229692 | 4.44E-04 |
| Gstk1 | -236.5712603 | 4.61E-04 |
| Mrpl2 | -85.54167277 | 4.66E-04 |
| Vhl | -10.84423982 | 4.77E-04 |
| Sh3bp4 | -262.6099894 | 5.09E-04 |
| Il4i1 | -24.3532674 | 5.13E-04 |
| Malsu1 | -44.63240884 | 5.34E-04 |
| Izumo4 | -225.4872653 | 5.49E-04 |
| Prr3 | -26.98370367 | 5.52E-04 |
| Blnk | -272.867583 | 5.54E-04 |
| Cercam | -330.7535762 | 5.60E-04 |
| Slc41a2 | -75.22813381 | 5.72E-04 |
| Ptprs | -76.32330451 | 5.82E-04 |
| Cd79b | -220.0489034 | 5.88E-04 |
| Tnfsf8 | -34.14954005 | 6.02E-04 |
| Creld1 | -33.64735806 | 6.24E-04 |
| Trp53i11 | -234.8139734 | 6.25E-04 |
| Plekhf1 | -225.8320141 | 6.28E-04 |
| Pdlim4 | -270.2681896 | 6.31E-04 |
| Adra2a | -219.8765678 | 6.93E-04 |
| Phc1 | -209.4703995 | 6.96E-04 |
| Wbscr27 | -43.27944908 | 6.97E-04 |
| Ppm1j | -276.3642687 | 7.04E-04 |
| Itgax | -7.072660094 | 7.44E-04 |
| Ttl | -217.0574317 | 7.76E-04 |
| Gfra2 | -194.8720995 | 7.86E-04 |
| Nenf | -35.84273866 | 8.22E-04 |
| Zkscan8 | -209.3448604 | 8.47E-04 |
| Gatm | -7.558331988 | 8.49E-04 |
| Fam109a | -266.4280996 | 8.67E-04 |
| Tesc | -197.1466786 | 9.02E-04 |
| 1600029D21Rik | -13.36793426 | 9.06E-04 |
| Hist1h2bc | -47.53650645 | 9.21E-04 |
| Cxx1b | -51.70102473 | 9.21E-04 |
| Rhoc | -16.13235225 | 9.36E-04 |
| Avpi1 | -238.3836037 | 9.37E-04 |
| Dusp3 | -37.38744034 | 9.69E-04 |
| Abi3bp | -240.5346953 | 1.00E-03 |
| Fezf2 | -259.5148147 | 1.00E-03 |
| Zbtb43 | -33.71359062 | 1.00E-03 |
| St3gal2 | -59.80410906 | 1.07E-03 |
| Zfyve21 | -236.8938225 | 1.11E-03 |
| Rel | -39.33727435 | 1.11E-03 |
| Igsf9 | -192.3753982 | 1.19E-03 |
| Ccdc80 | -11.43931553 | 1.20E-03 |
| Pdgfa | -60.3436966 | 1.20E-03 |
| Mfge8 | -9.492931761 | 1.23E-03 |
| Lpxn | -22.57431867 | 1.28E-03 |
| AW209491 | -67.63554581 | 1.34E-03 |
| Slc27a3 | -74.41367159 | 1.36E-03 |
| AA467197 | -7.497160471 | 1.38E-03 |
| Mrps23 | -14.11154246 | 1.39E-03 |
| Dcstamp | -16.53573515 | 1.47E-03 |
| Akr1e1 | -204.5268714 | 1.55E-03 |
| Ept1 | -24.72547422 | 1.60E-03 |
| Efna2 | -164.9361903 | 1.64E-03 |
| Dexi | -60.86673853 | 1.65E-03 |
| 4931406C07Rik | -21.30733762 | 1.67E-03 |
| Adck5 | -50.17783494 | 1.70E-03 |
| Lrrc42 | -216.8906461 | 1.72E-03 |
| St6gal1 | -356.086922 | 1.75E-03 |
| Haus3 | -41.62359142 | 1.75E-03 |
| Sccpdh | -157.2392301 | 1.78E-03 |
| Spire1 | -249.6050876 | 1.79E-03 |
| Prdx1 | -5.95117726 | 1.89E-03 |
| Ehd1 | -7.101413996 | 1.99E-03 |
| Lpcat1 | -31.37283196 | 2.05E-03 |
| Kif5c | -224.1168495 | 2.09E-03 |
| 1190002N15Rik | -145.4038843 | 2.18E-03 |
| Adat2 | -150.5131762 | 2.19E-03 |
| Ryr1 | -322.9009459 | 2.20E-03 |
| Catsperg1 | -188.6174908 | 2.22E-03 |
| Psd | -22.07689266 | 2.25E-03 |
| Hif3a | -175.5693306 | 2.32E-03 |
| D3Bwg0562e | -192.4939585 | 2.37E-03 |
| Marcks | -6.231030183 | 2.37E-03 |
| Cst3 | -5.698829898 | 2.41E-03 |
| Elk1 | -22.77720302 | 2.44E-03 |
| Wnt6 | -135.1548128 | 2.49E-03 |
| Mmd | -12.42970578 | 2.51E-03 |
| Foxo1 | -317.5956644 | 2.56E-03 |
| Ar | -137.5901039 | 2.60E-03 |
| Vmac | -149.9161714 | 2.63E-03 |
| Traf2 | -202.1211773 | 2.64E-03 |
| Gm16201 | -184.6825403 | 2.67E-03 |
| Calcoco1 | -215.070742 | 2.67E-03 |
| Mbd5 | -44.98297541 | 2.73E-03 |
| Vldlr | -363.0083138 | 2.80E-03 |
| Gm21986 | -37.75023591 | 2.80E-03 |
| Sestd1 | -29.94620076 | 2.81E-03 |
| Snap47 | -152.0965352 | 2.82E-03 |
| Tpm4 | -10.48784582 | 2.87E-03 |
| Clec10a | -51.07358719 | 2.88E-03 |
| Exo5 | -19.11581124 | 2.91E-03 |
| Usp20 | -132.853095 | 2.91E-03 |
| Atf3 | -6.624112925 | 3.05E-03 |
| Mtmr7 | -34.53004959 | 3.07E-03 |
| Gdpd1 | -159.245886 | 3.09E-03 |
| 4-Sep | -163.5034426 | 3.13E-03 |
| Caap1 | -61.58465222 | 3.17E-03 |
| Gm26735 | -288.1004721 | 3.25E-03 |
| Slc37a2 | -70.55014471 | 3.28E-03 |
| Smg6 | -19.08935149 | 3.41E-03 |
| Bmi1 | -24.46584112 | 3.45E-03 |
| Il2rb | -143.8083631 | 3.46E-03 |
| Gstt1 | -124.6892198 | 3.49E-03 |
| Ttll11 | -155.0743387 | 3.55E-03 |
| Gnb4 | -14.10549326 | 3.56E-03 |
| Klhl8 | -149.0637597 | 3.57E-03 |
| Fndc5 | -182.4273557 | 3.63E-03 |
| Nfkbia | -5.337182573 | 3.63E-03 |
| Dse | -39.27298068 | 3.67E-03 |
| Magee1 | -127.6804919 | 3.75E-03 |
| Kpna4 | -7.203524424 | 3.79E-03 |
| Bhlhe40 | -5.545984481 | 3.81E-03 |
| Amz2 | -20.08892191 | 3.83E-03 |
| Tsku | -118.5621642 | 3.84E-03 |
| Kdm3a | -19.24914096 | 3.86E-03 |
| Zbtb17 | -43.2212386 | 3.87E-03 |
| Nmb | -185.1450948 | 3.88E-03 |
| F730043M19Rik | -228.5501344 | 3.89E-03 |
| Socs2 | -5.368917829 | 4.07E-03 |
| Dcaf10 | -18.31683476 | 4.08E-03 |
| Wdsub1 | -240.0417452 | 4.10E-03 |
| Maoa | -47.86670551 | 4.13E-03 |
| Nfkb2 | -9.377940449 | 4.13E-03 |
| H2-M2 | -145.7691156 | 4.19E-03 |
| Serpinb6b | -9.842003676 | 4.20E-03 |
| Tmub1 | -21.97930906 | 4.21E-03 |
| Dnajc12 | -143.1720013 | 4.23E-03 |
| Rabgap1l | -12.06356134 | 4.29E-03 |
| Snx21 | -271.6022747 | 4.32E-03 |
| Egln3 | -5.76609916 | 4.37E-03 |
| Sesn2 | -22.95542424 | 4.41E-03 |
| Gimap6 | -124.3768096 | 4.44E-03 |
| Csrp2 | -142.173566 | 4.45E-03 |
| Sdc4 | -9.03286887 | 4.46E-03 |
| Klk1b11 | -7.041633924 | 4.46E-03 |
| Gprc5c | -123.2406589 | 4.54E-03 |
| Mllt3 | -26.92683682 | 4.60E-03 |
| Prkdc | -139.8382599 | 4.62E-03 |
| Fam49a | -15.61898025 | 4.70E-03 |
| Tpi1 | -4.65510173 | 4.70E-03 |
| Grasp | -233.0246201 | 4.77E-03 |
| Smtnl2 | -235.0005981 | 4.79E-03 |
| Mif | -5.047607824 | 4.90E-03 |
| Flnc | -13.99679893 | 4.93E-03 |
| Bnip3 | -5.744054757 | 4.94E-03 |
| Gm2a | -4.920455879 | 4.98E-03 |
| Pank1 | -65.16939181 | 5.14E-03 |
| Urb1 | -45.3294875 | 5.24E-03 |
| Dnah2 | -225.436198 | 5.25E-03 |
| Caprin2 | -154.2097556 | 5.25E-03 |
| Trp53i13 | -225.372608 | 5.26E-03 |
| Fam210b | -112.9579802 | 5.27E-03 |
| Slc12a4 | -141.0142837 | 5.31E-03 |
| Dpp4 | -141.6953684 | 5.34E-03 |
| Ephx1 | -18.60845602 | 5.35E-03 |
| Zbtb14 | -263.1814235 | 5.44E-03 |
| Atp9a | -40.13059952 | 5.53E-03 |
| Trmu | -131.0240078 | 5.54E-03 |
| Tex14 | -261.4203362 | 5.57E-03 |
| Mettl20 | -101.518282 | 5.58E-03 |
| F7 | -7.0232786 | 5.60E-03 |
| Gm8909 | -115.0065109 | 5.62E-03 |
| Pld4 | -6.128338151 | 5.64E-03 |
| Cd80 | -104.6182515 | 5.72E-03 |
| Nfkb1 | -4.942117628 | 5.80E-03 |
| Ndfip1 | -13.88321725 | 5.85E-03 |
| Vps25 | -7.257412862 | 5.87E-03 |
| Nags | -111.0324168 | 5.90E-03 |
| Siah1a | -158.0617784 | 5.91E-03 |
| D14Abb1e | -34.48670095 | 5.95E-03 |
| Rpap2 | -107.4777206 | 6.01E-03 |
| Rbpms | -26.99732139 | 6.01E-03 |
| Tmem37 | -22.86794553 | 6.05E-03 |
| Trip10 | -23.45382135 | 6.06E-03 |
| Nphp1 | -179.5312096 | 6.11E-03 |
| Trit1 | -37.93887531 | 6.11E-03 |
| Homez | -153.3198335 | 6.13E-03 |
| Stat4 | -25.2214953 | 6.17E-03 |
| Serpinb9b | -18.92733142 | 6.22E-03 |
| Nipa1 | -103.1304921 | 6.24E-03 |
| Tnfrsf13c | -225.4278523 | 6.32E-03 |
| Bcl2a1d | -7.31535647 | 6.40E-03 |
| Sema6d | -266.0060062 | 6.43E-03 |
| E2f5 | -95.15752972 | 6.50E-03 |
| Slc6a12 | -17.48162933 | 6.51E-03 |
| D10Jhu81e | -11.70454282 | 6.53E-03 |
| Serpinb6a | -10.91958577 | 6.56E-03 |
| Nudt2 | -162.1951455 | 6.56E-03 |
| Mink1 | -10.8467841 | 6.67E-03 |
| St3gal3 | -13.40312356 | 6.71E-03 |
| Zfp772 | -102.9246083 | 6.72E-03 |
| Eva1b | -28.79314756 | 6.73E-03 |
| Nfe2l1 | -7.909523406 | 6.79E-03 |
| Ear1 | -17.68472317 | 6.91E-03 |
| Zfp397 | -142.0256102 | 6.95E-03 |
| Cdkn2b | -14.03505641 | 7.00E-03 |
| Rnf19b | -4.681761693 | 7.32E-03 |
| Zfp874a | -57.56363493 | 7.37E-03 |
| Gm15327 | -104.9413622 | 7.37E-03 |
| Sigirr | -37.79778803 | 7.47E-03 |
| Cdpf1 | -90.26975976 | 7.56E-03 |
| Afp | -225.9298339 | 7.81E-03 |
| Clec7a | -4.623219769 | 7.90E-03 |
| Zfp28 | -32.37423822 | 7.91E-03 |
| Ptms | -5.594979917 | 7.96E-03 |
| Pkp3 | -28.41043488 | 8.01E-03 |
| 2700060E02Rik | -8.326900555 | 8.02E-03 |
| Lrrk1 | -5.581258616 | 8.06E-03 |
| Dis3l2 | -104.2551453 | 8.08E-03 |
| Nudt14 | -17.37737728 | 8.12E-03 |
| Cdon | -220.9653107 | 8.13E-03 |
| Alkbh7 | -213.0346698 | 8.18E-03 |
| Ccl11 | -107.4580316 | 8.19E-03 |
| Wbp5 | -13.44609899 | 8.22E-03 |
| Jmy | -40.58638199 | 8.22E-03 |
| H2-Aa | -4.344161511 | 8.24E-03 |
| Acot7 | -7.899727018 | 8.24E-03 |
| Mycl | -118.7642941 | 8.25E-03 |
| 2610034B18Rik | -208.7206344 | 8.28E-03 |
| Selenbp1 | -15.18702589 | 8.31E-03 |
| Atf5 | -9.452535903 | 8.32E-03 |
| Dctn4 | -6.288478828 | 8.33E-03 |
| Clec1b | -176.957105 | 8.43E-03 |
| Phkg2 | -8.719691038 | 8.44E-03 |
| Sox4 | -15.63567144 | 8.52E-03 |
| Nrn1 | -251.8495694 | 8.56E-03 |
| Tceal8 | -12.04751313 | 8.57E-03 |
| Mthfd2 | -5.869962866 | 8.59E-03 |
| Inhba | -7.754741293 | 8.75E-03 |
| Gm5148 | -8.239562176 | 8.77E-03 |
| Cpt2 | -95.19575411 | 8.78E-03 |
| Kdm1b | -88.34456694 | 8.79E-03 |
| Slc1a4 | -100.0553548 | 8.91E-03 |
| Grap | -191.8403132 | 9.04E-03 |
| Car2 | -18.67755933 | 9.05E-03 |
| Vps13a | -114.7952557 | 9.05E-03 |
| Alpk2 | -108.0281753 | 9.11E-03 |
| Pwwp2a | -24.64794355 | 9.25E-03 |
| Suv420h1 | -38.04864804 | 9.28E-03 |
| Nudt17 | -57.45238571 | 9.35E-03 |
| Dpysl5 | -28.95914739 | 9.36E-03 |
| Zfml | -9.126329273 | 9.59E-03 |
| Gpi1 | -4.243424972 | 9.62E-03 |
| Tes | -6.13754987 | 9.69E-03 |
| Jmjd4 | -35.37274143 | 9.95E-03 |
| Bcl2a1b | -5.542959404 | 0.010024647 |
| Kat8 | -193.1253732 | 0.010180655 |
| Bbc3 | -20.70329594 | 0.010231921 |
| Top3a | -90.82650007 | 0.010431817 |
| Tnfrsf18 | -22.67606615 | 0.010435983 |
| Ahnak | -4.95535701 | 0.010449742 |
| Haus8 | -10.30817697 | 0.010471269 |
| Rasgef1b | -158.1532868 | 0.01048751 |
| Cx3cl1 | -159.2691621 | 0.010517753 |
| Pdcd1lg2 | -6.143225413 | 0.010533079 |
| Mical3_1 | -35.00435152 | 0.010534965 |
| Herc1 | -8.552557539 | 0.010544632 |
| Tcea3 | -197.9832097 | 0.010553662 |
| Mzt2 | -30.4359115 | 0.010663201 |
| Rras | -12.79258027 | 0.010672609 |
| Lace1 | -78.04004242 | 0.010753414 |
| Adk | -13.10010741 | 0.010801324 |
| Six1 | -55.32847712 | 0.010935431 |
| Ears2 | -14.29069639 | 0.011018016 |
| Myo1b | -116.9194344 | 0.01108579 |
| Ptk6 | -191.7352064 | 0.011153825 |
| Vcan | -11.91369113 | 0.011254525 |
| Gm26782 | -202.5772521 | 0.011258522 |
| Chchd6 | -98.69412171 | 0.01128081 |
| Cldn1 | -20.30533132 | 0.011308884 |
| Arl13b | -66.18607011 | 0.011313211 |
| Clic4 | -4.19850416 | 0.011488568 |
| Rnf152 | -126.7854881 | 0.011489652 |
| H2-Ab1 | -4.154390877 | 0.011501597 |
| Mmp9 | -9.41908655 | 0.011526676 |
| Rftn1 | -5.641765589 | 0.01153914 |
| Thra | -37.30254526 | 0.011691249 |
| Icam1 | -4.950567787 | 0.011727103 |
| Lonrf3 | -96.89909752 | 0.01181823 |
| Syn1 | -96.43088065 | 0.011972922 |
| Tmcc3 | -10.96643172 | 0.012100427 |
| Ptov1 | -9.284720993 | 0.012140873 |
| Bad | -12.07798481 | 0.012164362 |
| Hist1h2ae | -180.9045003 | 0.012202962 |
| Gm20459 | -26.80198232 | 0.012372615 |
| Ccl9 | -4.383911399 | 0.012447446 |
| Alg9 | -12.32887588 | 0.012558554 |
| Sfmbt2 | -142.3115106 | 0.01258677 |
| Lrrc8c | -8.09069157 | 0.012597095 |
| Ltbp3 | -11.08015956 | 0.012694596 |
| Hmgn3 | -93.86395338 | 0.012746608 |
| Oaz2 | -18.36886364 | 0.012757123 |
| Gm16845 | -39.83128314 | 0.012833914 |
| Sox5 | -145.3011988 | 0.012917054 |
| Snx7 | -145.3011007 | 0.012922161 |
| Ccr2 | -5.34681927 | 0.013051357 |
| Gm21839 | -26.59417901 | 0.01306375 |
| Dpys | -161.1465783 | 0.013074724 |
| Zfp385a | -9.952725263 | 0.013115456 |
| 4930556M19Rik_1 | -173.0417963 | 0.013256117 |
| Zfat | -127.9754849 | 0.013266706 |
| Parvb | -28.28017545 | 0.013296987 |
| Ppp1r13b | -13.68265501 | 0.013345319 |
| Speg | -11.36636308 | 0.01335492 |
| Gm26667 | -143.838006 | 0.013418457 |
| Ankrd55 | -145.2208623 | 0.013420292 |
| Capn5 | -101.3691801 | 0.013429773 |
| Zfp386 | -21.10425299 | 0.013451785 |
| Mpzl2 | -98.06357237 | 0.013484846 |
| Idh1 | -5.638472967 | 0.01353625 |
| Fam221a | -196.4556804 | 0.01356153 |
| Zfp707 | -29.07873028 | 0.013639455 |
| Dgcr8 | -12.70385025 | 0.013690097 |
| Cnn3 | -7.353661967 | 0.013768717 |
| Trim7 | -73.18542431 | 0.013840187 |
| Gtf3c3 | -111.7619881 | 0.013929544 |
| Cgref1 | -128.7235236 | 0.013937996 |
| S1pr3 | -25.21979447 | 0.014097842 |
| Nr1i3 | -19.34997501 | 0.01413379 |
| Basp1 | -3.781068237 | 0.0141419 |
| Taf15 | -6.322060564 | 0.014214017 |
| Adcy9 | -146.5569071 | 0.014223868 |
| Calhm2 | -75.73088736 | 0.014256929 |
| Azi1 | -151.3267339 | 0.014300531 |
| Eno3 | -13.09880908 | 0.014351757 |
| Ppp1r3b | -11.85660844 | 0.01474382 |
| Copz2 | -111.6977713 | 0.014753497 |
| H2-Ea-ps | -3.887599734 | 0.014841098 |
| Plxna1 | -125.8629203 | 0.014873648 |
| Akap10 | -12.0116626 | 0.014908062 |
| Ldlrad3 | -131.5400871 | 0.014935592 |
| Klra2 | -13.51290361 | 0.014962737 |
| Pex26 | -14.0259708 | 0.014988644 |
| Tmem107 | -26.66654613 | 0.015063813 |
| Gm11634 | -145.5077237 | 0.015133006 |
| Ankrd52 | -101.3370415 | 0.015228351 |
| Pdcd7 | -138.4094819 | 0.015269414 |
| Slc25a32 | -41.8743358 | 0.01531135 |
| Gpr126 | -148.4417457 | 0.015617637 |
| Plekhm2 | -6.952824084 | 0.015647831 |
| Mtmr9 | -97.95714867 | 0.01571014 |
| Lrrc20 | -154.6701653 | 0.015771846 |
| Cxx1c | -82.54373566 | 0.015789803 |
| Tnfaip3 | -6.208892972 | 0.015805296 |
| Spice1 | -26.85831138 | 0.015981953 |
| Ptpn4 | -119.5257265 | 0.016140855 |
| Mdc1 | -19.0204486 | 0.016160677 |
| Phf6 | -13.6610661 | 0.016190498 |
| B930041F14Rik_1 | -157.5696555 | 0.01621156 |
| Arhgap29 | -28.91317458 | 0.016369526 |
| Sft2d2 | -4.980189905 | 0.016375302 |
| Lzts2 | -24.54335685 | 0.016436022 |
| Wrb | -123.7859902 | 0.016447331 |
| Cd40 | -12.08635939 | 0.016525134 |
| Prkab2 | -122.3232666 | 0.016565376 |
| H2-DMb2 | -5.342493231 | 0.016565729 |
| Stx1b | -126.4903313 | 0.016579053 |
| Acbd4 | -27.2585115 | 0.016582476 |
| Cdkn2a | -9.731104266 | 0.016646376 |
| Abcc1 | -7.844657394 | 0.016747516 |
| Gramd3 | -140.8443316 | 0.016756829 |
| Tmem241 | -123.273515 | 0.016771668 |
| Cox4i1 | -4.763741469 | 0.016802471 |
| Pde4dip | -20.3007736 | 0.01681288 |
| Elovl7 | -131.4657971 | 0.016826238 |
| Ntn4 | -67.86762808 | 0.016883816 |
| Il31ra | -204.3673113 | 0.016984961 |
| Slc5a6 | -127.5850074 | 0.017025985 |
| Dusp5 | -5.953281203 | 0.017112857 |
| Ttc21b | -20.19656384 | 0.017203196 |
| Micu3 | -128.389443 | 0.017238538 |
| Nov | -7.209774295 | 0.017379343 |
| Btg1 | -3.639787617 | 0.017411845 |
| Pfkp | -3.792044216 | 0.01744738 |
| Farp1 | -36.90250819 | 0.0174997 |
| A830035O19Rik | -133.5411526 | 0.017560453 |
| Gpr132 | -14.27899668 | 0.017660738 |
| Gm12606 | -163.02055 | 0.017754466 |
| Nipal1 | -123.7073343 | 0.017772802 |
| Dyrk1b | -75.46712882 | 0.017890272 |
| Tnfrsf12a | -64.28772938 | 0.017890625 |
| C230037L18Rik | -119.0263026 | 0.018044554 |
| Tango6 | -182.0904444 | 0.018105421 |
| BC064078 | -78.33042157 | 0.018136116 |
| Lyrm5 | -24.1392962 | 0.018253778 |
| Dnajb4 | -13.75424665 | 0.018337008 |
| Rdm1 | -127.8733409 | 0.018366885 |
| Dctn5 | -24.20681167 | 0.018659045 |
| Gm26992 | -148.2709853 | 0.018674877 |
| Capg | -3.617374053 | 0.018727231 |
| Bcl2l14 | -89.67765353 | 0.018845148 |
| Plxnd1 | -7.822277592 | 0.018917225 |
| Hmox1 | -10.16019526 | 0.018919909 |
| 5430435G22Rik | -27.36930128 | 0.018954272 |
| Ccbl1 | -127.6630341 | 0.018997838 |
| Tia1 | -9.426837462 | 0.01905531 |
| Ythdc2 | -106.8511136 | 0.019128308 |
| Zfp46 | -82.27754713 | 0.01916476 |
| Pmaip1 | -5.163086798 | 0.019265031 |
| Tspyl3 | -67.62285792 | 0.019529788 |
| Apln | -115.9208885 | 0.019626281 |
| Fabp4 | -14.53739475 | 0.019629102 |
| Ncdn | -15.22597544 | 0.019754896 |
| Ccdc91 | -65.53281192 | 0.01978705 |
| Tmem176a | -8.467643351 | 0.01982919 |
| Npr1 | -51.98196148 | 0.019833111 |
| Gsn | -3.945892528 | 0.019841271 |
| Mtrf1 | -192.8151682 | 0.019913175 |
| Tnfaip8l1 | -120.2554299 | 0.020017736 |
| Rai14 | -6.847738849 | 0.020165649 |
| Slc12a2 | -12.32590467 | 0.020213335 |
| Plcd1 | -107.1267652 | 0.020272413 |
| F630040K05Rik | -106.7524144 | 0.020370938 |
| Crocc | -31.19765622 | 0.020435353 |
| M1ap | -94.79698721 | 0.020442317 |
| Pigg | -130.792075 | 0.020448043 |
| Seh1l | -6.970246007 | 0.020529164 |
| Mllt6 | -4.440656815 | 0.020643372 |
| Cacnb4 | -140.1941628 | 0.020828931 |
| Casz1 | -6.653550161 | 0.0208846 |
| Cmtm3 | -8.172268389 | 0.020896635 |
| Mt2 | -3.871729101 | 0.020910898 |
| Dapk3 | -12.2569173 | 0.020956439 |
| Sox12 | -110.2370154 | 0.020981019 |
| Hr | -106.3239113 | 0.021137021 |
| Kbtbd13 | -115.5792073 | 0.021728972 |
| Klf16 | -105.2231039 | 0.021912805 |
| Prkcq | -38.42140175 | 0.021994901 |
| Art4 | -103.4032803 | 0.021996476 |
| Chi3l3 | -3.352791871 | 0.022091395 |
| Daam1 | -17.42628838 | 0.022206471 |
| Plin3 | -4.38814878 | 0.022210329 |
| Csf1 | -3.708756297 | 0.022251216 |
| Tmem206 | -31.75206151 | 0.022438964 |
| Cdkn1a | -3.548841044 | 0.022450457 |
| Sdcbp2 | -112.6667985 | 0.022552285 |
| Scd1 | -9.273241743 | 0.022618778 |
| Bivm | -108.9830523 | 0.022622843 |
| Gm15051 | -33.64964007 | 0.022648925 |
| Slc7a11 | -4.9416078 | 0.022858724 |
| Erp27 | -125.9513797 | 0.022861013 |
| Nrp2 | -5.091162293 | 0.023003852 |
| Apbb2 | -9.795955404 | 0.02307943 |
| Gm340 | -109.8466038 | 0.023352274 |
| Arc | -25.75268981 | 0.023379169 |
| RP24-325L16.1 | -106.7749697 | 0.023674711 |
| Hist1h1c | -4.177764806 | 0.023677699 |
| Marcksl1 | -4.507095987 | 0.023714517 |
| Sepw1 | -5.438602423 | 0.023782625 |
| Parp3 | -8.858836785 | 0.02396162 |
| Acvrl1 | -15.50535335 | 0.023988473 |
| Smad3 | -98.09954251 | 0.02417019 |
| Rab26 | -97.83138745 | 0.024291941 |
| Cpm | -131.0201326 | 0.024360253 |
| Araf | -4.608252666 | 0.024464829 |
| Mxd4 | -8.355712837 | 0.024825944 |
| Stxbp6 | -139.1430604 | 0.024858255 |
| Tmem120a | -10.87575271 | 0.024867068 |
| 4930506C21Rik | -86.33685677 | 0.024886219 |
| Piwil4 | -95.03451589 | 0.024967038 |
| Tk2 | -9.856084389 | 0.025124834 |
| Tmtc2 | -103.9308532 | 0.025168577 |
| Ung | -17.47286481 | 0.025273181 |
| Trappc9 | -11.73392977 | 0.025327125 |
| Med12l | -19.7865678 | 0.025387439 |
| Gm7722 | -11.56683328 | 0.02548121 |
| Naa35 | -5.333731939 | 0.025534013 |
| Gm26631 | -12.45526224 | 0.025903809 |
| Btbd2 | -8.656840582 | 0.026180142 |
| Rpl13-ps3 | -8.265079767 | 0.026279978 |
| Nudt21 | -5.147161385 | 0.026488223 |
| Supt3 | -91.56932292 | 0.026541055 |
| Pstk | -122.9168461 | 0.02657949 |
| Zfp69 | -112.4309063 | 0.02662595 |
| Trim3 | -12.27053566 | 0.02672239 |
| Pea15a | -5.797598914 | 0.02675876 |
| Vat1 | -5.748045639 | 0.02699638 |
| Aqp9 | -5.068634258 | 0.027093707 |
| Fosl1 | -124.6932718 | 0.027130085 |
| Aplp2 | -4.322293843 | 0.027325622 |
| Map9 | -74.24117145 | 0.027434024 |
| Gm20458 | -9.191991435 | 0.027547715 |
| Tmem126b | -112.2738523 | 0.027587784 |
| Mical2 | -10.47493078 | 0.027764602 |
| Zfp771 | -13.39348689 | 0.027837071 |
| Rhobtb3 | -148.1162439 | 0.027841539 |
| Mab21l3 | -110.1342403 | 0.027924498 |
| Spsb4 | -113.0522791 | 0.02798675 |
| Panx1 | -33.49730016 | 0.028003419 |
| Wars | -4.581596213 | 0.028212108 |
| B230217C12Rik_2 | -64.2052183 | 0.02824257 |
| Gm3650 | -19.55661129 | 0.028270348 |
| Cacnb1 | -102.1873275 | 0.028524055 |
| Calcrl | -131.1874698 | 0.028551374 |
| Ado | -6.442199107 | 0.028551552 |
| Msto1 | -9.606400401 | 0.02881213 |
| 1810030O07Rik | -4.546404262 | 0.028844183 |
| Dynlt3 | -6.127441761 | 0.028868459 |
| Usp6nl | -17.91378916 | 0.028936974 |
| Zc3h10 | -9.432634746 | 0.029122575 |
| Selp | -97.38019221 | 0.029123499 |
| Kifap3 | -99.38422462 | 0.029201168 |
| Rbm45 | -5.386654037 | 0.029405266 |
| Mrps12 | -5.514627846 | 0.029413765 |
| Rps26 | -3.574585979 | 0.02963591 |
| Nagk | -5.541371068 | 0.029665603 |
| Arfip2 | -10.23340092 | 0.02968063 |
| Slc16a3 | -3.778128661 | 0.029700128 |
| Tcf4 | -4.052347312 | 0.029739678 |
| Rbpj | -3.34831316 | 0.030078909 |
| Gm7589 | -4.321962902 | 0.030079383 |
| Dmrta2 | -84.50869288 | 0.03008243 |
| Prss36 | -103.7968191 | 0.030110271 |
| Cxcl11 | -101.471671 | 0.030153296 |
| Atp6v1h | -8.684495981 | 0.030165937 |
| 2210404O07Rik | -7.983462189 | 0.030282661 |
| Gas2l1 | -7.372440968 | 0.030283073 |
| Slc2a3 | -7.780404287 | 0.030378623 |
| Hbegf | -7.896989375 | 0.030420271 |
| Nt5c | -4.882673903 | 0.03043486 |
| Nhlrc1 | -86.10235122 | 0.030487836 |
| Slc41a1 | -20.72029893 | 0.030682688 |
| Kif1a | -18.0857656 | 0.030689092 |
| C330018D20Rik | -15.05603793 | 0.030789869 |
| Treml4 | -102.0615862 | 0.030801597 |
| Myl6 | -3.662725808 | 0.030802353 |
| Sik1 | -35.09502637 | 0.030839152 |
| D2hgdh | -8.832021926 | 0.030914073 |
| Rasgrp1 | -97.45322904 | 0.031100553 |
| R3hcc1 | -60.44098172 | 0.031148449 |
| Ypel1 | -55.99787538 | 0.031518774 |
| Adam8 | -3.867851658 | 0.031641086 |
| Lgals3 | -3.25620849 | 0.031666677 |
| D730003I15Rik | -204.5786487 | 0.031711264 |
| Gabarapl1 | -6.323376922 | 0.031793027 |
| Pip5k1c | -3.625524345 | 0.032055785 |
| Pdzk1ip1 | -85.55539635 | 0.03205968 |
| Npr2 | -106.0585683 | 0.032062802 |
| Prkcd | -3.317490175 | 0.032137015 |
| Slc4a8 | -73.0146446 | 0.032224371 |
| Bcl2l11 | -4.951349984 | 0.032253106 |
| Acads | -10.50342826 | 0.032335112 |
| Fam83h | -96.31249162 | 0.032420743 |
| Wdr54 | -120.7246065 | 0.032439742 |
| Clec2d | -12.47498867 | 0.032477714 |
| Il27ra | -80.89960027 | 0.032574736 |
| Agpat4 | -8.696306133 | 0.032699983 |
| Slc39a6 | -13.84981287 | 0.032717377 |
| Ankrd13c | -70.40250532 | 0.032806936 |
| Plbd1 | -5.582220933 | 0.032841415 |
| Smurf1 | -7.349002176 | 0.032890062 |
| Macrod1 | -79.59849233 | 0.033043156 |
| Fam221b | -4.830935731 | 0.033129513 |
| Tnfrsf10b | -11.27572549 | 0.033156645 |
| Gm26582 | -9.401078438 | 0.033160478 |
| Capn3 | -81.5219805 | 0.03318678 |
| Col5a1 | -17.57575263 | 0.033201604 |
| Nos2 | -4.250887506 | 0.03322301 |
| Phactr1 | -87.27345698 | 0.033344955 |
| Btf3 | -3.581070555 | 0.033356662 |
| Trim29 | -19.6433356 | 0.033407968 |
| Armc10 | -5.969552524 | 0.033659071 |
| Ly86 | -79.30813531 | 0.03374257 |
| Slc6a15 | -124.8914266 | 0.0337538 |
| Selm | -21.21834011 | 0.033968834 |
| Cd70 | -77.83955857 | 0.033990894 |
| Gm20603 | -9.686727287 | 0.034011474 |
| Il11ra1 | -11.37846796 | 0.034066246 |
| Dcaf4 | -107.3022097 | 0.034237627 |
| Taf9b | -109.3391405 | 0.034319771 |
| Sema4c | -52.57414737 | 0.034508461 |
| Cbln3 | -60.82286379 | 0.034584434 |
| Prdx2 | -3.865282561 | 0.034680924 |
| Zfp748 | -90.72546271 | 0.034734209 |
| Capn7 | -141.5343003 | 0.034738161 |
| Recql | -8.68154781 | 0.03480385 |
| Mt3 | -77.95929363 | 0.034917564 |
| Rpl17 | -4.852449001 | 0.034999346 |
| Tm9sf1 | -12.95777422 | 0.035010844 |
| Gm9726 | -42.45189813 | 0.035095301 |
| Mtfr1 | -15.8274306 | 0.035308867 |
| Carns1 | -110.3752785 | 0.035582612 |
| Hps5 | -10.08531118 | 0.035668122 |
| 9330175E14Rik | -14.32182567 | 0.035741357 |
| Rabl3 | -24.13782277 | 0.035770277 |
| Fbxo8 | -28.69421208 | 0.035782607 |
| Asb4 | -146.3900244 | 0.03592028 |
| Psme2 | -3.434900928 | 0.036045864 |
| Gm14597 | -72.24408818 | 0.036337218 |
| Rpl28 | -3.681899576 | 0.03637516 |
| Rcor2 | -75.69555641 | 0.036538793 |
| Mall | -19.22282552 | 0.036584556 |
| Thumpd1 | -9.451340383 | 0.036656528 |
| Fbxw5 | -5.52172626 | 0.036850419 |
| Klk1b3 | -110.9293331 | 0.036946318 |
| Fbxo4 | -9.653263753 | 0.036972158 |
| Ccdc85b | -73.19569962 | 0.037101459 |
| Ndrg2 | -6.24023496 | 0.037287476 |
| Ddx18 | -4.337600754 | 0.037328576 |
| Ifrd1 | -5.160750023 | 0.037396555 |
| Suco | -11.05103054 | 0.037469893 |
| Laptm4b | -85.12386309 | 0.037488209 |
| Gimap5 | -82.0656458 | 0.03753847 |
| Rbfox2 | -14.50233411 | 0.037625537 |
| Zbtb49 | -243.9566823 | 0.03763588 |
| Pole2 | -48.51502898 | 0.037687112 |
| Macrod2 | -116.7937315 | 0.037688 |
| Fam78b | -28.03737969 | 0.037969227 |
| Dbndd2 | -16.18047143 | 0.038026284 |
| Dhdds | -5.075616469 | 0.038279898 |
| 4933427D14Rik | -25.35042813 | 0.038296136 |
| Slc6a13 | -12.38329271 | 0.038308547 |
| Atrip | -6.511064832 | 0.03851811 |
| Plxdc2 | -3.721848832 | 0.038542597 |
| Cp | -9.354903658 | 0.038741304 |
| Anxa6 | -4.085001153 | 0.038791656 |
| Ass1 | -4.315984258 | 0.038847223 |
| Rnf14 | -6.67584237 | 0.03888218 |
| Mbtd1 | -3.853531552 | 0.038954646 |
| Gucy1a3 | -84.24484982 | 0.03911424 |
| Nrxn2 | -70.14325958 | 0.039156584 |
| Mblac2 | -70.93623619 | 0.039169643 |
| 1110002J07Rik | -74.13587545 | 0.039249049 |
| Gp5 | -72.75250683 | 0.039320163 |
| Uevld | -61.86546382 | 0.03935864 |
| Apol10b | -42.99420454 | 0.039393456 |
| Krt10 | -79.67176369 | 0.039395102 |
| Clmn | -83.65700089 | 0.039556941 |
| Ctsz | -3.287645194 | 0.039695333 |
| Rbbp9 | -73.54600462 | 0.039795222 |
| Gga1 | -4.039913955 | 0.040244523 |
| Fgf13 | -167.3345151 | 0.040244792 |
| Icos | -10.95394499 | 0.040349314 |
| Adamts7 | -167.9840966 | 0.040377952 |
| Ip6k2 | -46.72039001 | 0.040411232 |
| Aebp2 | -3.570304391 | 0.040543733 |
| Pafah1b3 | -4.583523413 | 0.040774191 |
| Cldn25 | -4.864252795 | 0.040913135 |
| Lysmd3 | -140.9051696 | 0.04098612 |
| Frzb | -140.9053571 | 0.041000296 |
| Rplp1 | -3.614870064 | 0.041045636 |
| Rps9 | -3.480783383 | 0.041189287 |
| Pgm3 | -26.9407756 | 0.041213991 |
| Ctdp1 | -7.186856018 | 0.041346259 |
| Hace1 | -13.33167229 | 0.041347561 |
| 6030442K20Rik | -46.76905884 | 0.041452814 |
| Vwa5a | -3.57441846 | 0.041559512 |
| Pfdn5 | -3.895042447 | 0.041702 |
| Stard3nl | -4.882057121 | 0.041842869 |
| Arhgef28 | -84.21511731 | 0.041912509 |
| Zmiz2 | -4.803030882 | 0.04192309 |
| Ikzf4 | -6.432402673 | 0.041929856 |
| Edn1 | -4.986984605 | 0.041942898 |
| Eid2 | -17.89420594 | 0.042149698 |
| Cc2d2a | -88.01595662 | 0.042340619 |
| Pmm2 | -4.92748474 | 0.042702688 |
| Tmem106c | -73.17294633 | 0.042785773 |
| Plk2 | -4.988712818 | 0.042787102 |
| Pcsk4 | -68.90476422 | 0.042819658 |
| BC035044 | -12.99753844 | 0.042833328 |
| Ube2l6 | -5.072504783 | 0.042865779 |
| Fam174b | -40.64204036 | 0.042866854 |
| Olfm1 | -9.163666204 | 0.042888039 |
| Ak2 | -3.165004139 | 0.042891112 |
| Nudcd2 | -4.946559613 | 0.042916906 |
| Tnfsf4 | -26.37175629 | 0.042997852 |
| Zfp93 | -158.2336131 | 0.043024547 |
| Rps3a1 | -3.269247563 | 0.043062987 |
| Ccdc34 | -4.595815519 | 0.043321579 |
| Usp35 | -83.79088196 | 0.043392575 |
| Pvrl3 | -85.24551439 | 0.043401026 |
| Ptpn22 | -94.45382789 | 0.0435567 |
| Mybpc3 | -31.26214163 | 0.04362049 |
| Cisd3 | -21.07288673 | 0.043640002 |
| Gltp | -3.664884367 | 0.04364143 |
| Otub2 | -75.77934072 | 0.043687025 |
| Rhobtb1 | -76.83630458 | 0.043763522 |
| Rnf43 | -74.60957026 | 0.04383246 |
| Rnmtl1 | -66.75191609 | 0.04399981 |
| Prickle1 | -122.0077442 | 0.04403964 |
| Irf5 | -3.156009099 | 0.044566369 |
| Ppp2r3d | -65.06226294 | 0.044758829 |
| Gm807 | -94.54964859 | 0.044946654 |
| Uqcr10 | -3.633060018 | 0.044969994 |
| Spred2 | -6.033524737 | 0.044971348 |
| Gm20429 | -153.6827322 | 0.045064546 |
| Nub1 | -4.676405069 | 0.045106057 |
| Map4k1 | -8.290896637 | 0.045117384 |
| Ccdc90b | -13.83667419 | 0.045120009 |
| Dcaf11 | -11.36554235 | 0.045192356 |
| Crcp | -9.682810699 | 0.045275868 |
| Myo1e | -4.36918603 | 0.045322684 |
| Vma21-ps | -43.94789922 | 0.045420126 |
| Vsig8 | -135.6976901 | 0.045446477 |
| Galnt9 | -70.70893847 | 0.045660407 |
| Hcls1 | -3.281623488 | 0.045715416 |
| Klrb1a | -52.96001484 | 0.046180124 |
| Fam26f | -39.97157578 | 0.046180351 |
| Gm15050 | -98.24572301 | 0.046319009 |
| Pcnx | -10.39238383 | 0.046349815 |
| Evc2 | -128.7147132 | 0.046444081 |
| Atp11a | -12.47966706 | 0.04653872 |
| Igsf3 | -90.18764714 | 0.046583628 |
| Egr2 | -6.797680811 | 0.04684086 |
| Iba57 | -71.20762383 | 0.04710993 |
| Cradd | -14.5058596 | 0.047188637 |
| Hfe | -7.501568283 | 0.047497861 |
| Ahsa2 | -9.106974898 | 0.047564359 |
| L1cam | -9.378136159 | 0.047587241 |
| Trib3 | -7.227324392 | 0.047666211 |
| Bcorl1 | -7.403804019 | 0.047832232 |
| Mms22l | -61.33152059 | 0.047958276 |
| Cox7a2l | -3.143246804 | 0.048248479 |
| Klhl3 | -60.31696419 | 0.04835511 |
| Bod1 | -7.044309772 | 0.048387161 |
| Rbm12b1 | -60.64859127 | 0.048467196 |
| Mcf2l | -8.261369035 | 0.048541823 |
| Slc25a46 | -5.538763775 | 0.048581386 |
| Arl5c | -3.890972313 | 0.048602192 |
| Tbc1d4 | -5.797522481 | 0.048701062 |
| Asns | -6.268143261 | 0.049001679 |
| Trim46 | -66.96333545 | 0.049074304 |
| Chrnb1 | -73.88109611 | 0.04913831 |
| Slc25a4 | -3.15968808 | 0.04926991 |
| Guk1 | -3.549212153 | 0.049301484 |
| Ehhadh | -39.23836277 | 0.049311534 |
| Snx9 | -8.577073187 | 0.049380138 |
| Mgll | -3.200048622 | 0.049416708 |
| Asah2 | -62.224529 | 0.049433295 |
| Nupr1 | -6.722584093 | 0.049529727 |
| Dusp2 | -29.652793 | 0.049555479 |
| Gm7353 | -19.57247609 | 0.049590408 |
| Ric8 | -4.330118681 | 0.049605539 |
| BC147527 | -142.3059272 | 0.049684566 |
| Arl6 | -90.69732296 | 0.049839714 |
| Fam219a | -60.750395 | 0.049885385 |

***Up regulated genes***

| *Gene* | log2 fold change | P-value |
| --- | --- | --- |
| Mogat2 | 1079.701575 | 3.18E-07 |
| Dgkg | 897.2041552 | 1.56E-05 |
| Map3k5 | 822.7477864 | 2.07E-06 |
| Mcpt1 | 749.6499032 | 1.18E-15 |
| BC117090 | 560.114552 | 9.52E-13 |
| Mcpt2 | 482.949091 | 1.49E-11 |
| BC094916 | 471.5143405 | 1.97E-04 |
| Sass6 | 380.7166978 | 2.88E-04 |
| Homer2 | 371.5000837 | 3.54E-04 |
| Cenpo | 371.3923852 | 1.45E-04 |
| Tcrg-C1 | 350.0082895 | 2.95E-03 |
| Gpc3 | 322.1182367 | 3.72E-04 |
| Klrb1f | 319.1938389 | 1.22E-03 |
| Dtx2 | 315.5525757 | 9.61E-03 |
| 5430421N21Rik | 301.3742602 | 6.81E-04 |
| Nlrp6 | 291.7102112 | 3.52E-03 |
| Ceacam10 | 281.3546334 | 2.67E-06 |
| Tph1 | 271.9578172 | 3.70E-04 |
| Ltb4r2 | 264.6699426 | 6.24E-03 |
| Nupl2 | 262.3229003 | 5.42E-03 |
| Ugt8a | 256.2995663 | 4.34E-04 |
| Clnk | 239.3644785 | 9.00E-04 |
| Muc13 | 235.6787893 | 3.43E-03 |
| Agpat2 | 235.3319332 | 3.16E-03 |
| Tpsab1 | 234.7770372 | 8.19E-04 |
| Mctp2 | 233.2719123 | 8.31E-03 |
| Gca | 232.8132868 | 1.80E-03 |
| Ccdc88c | 228.5878353 | 6.21E-03 |
| Oxsm | 226.2112623 | 0.02357402 |
| Pik3ap1 | 223.5100028 | 6.84E-03 |
| Dkkl1 | 223.5043041 | 7.20E-04 |
| Rnf144b | 223.0004444 | 9.13E-03 |
| Fcer1a | 222.6508056 | 8.97E-08 |
| Cluap1 | 222.485539 | 7.28E-04 |
| Nrip3 | 220.6528796 | 7.09E-03 |
| Acad11 | 218.8186498 | 0.045931527 |
| Adal | 187.6289802 | 0.010389191 |
| Sigmar1 | 183.6423395 | 6.34E-03 |
| Tnfsf14 | 181.5341134 | 3.12E-03 |
| Rnf125 | 176.0216175 | 0.015722814 |
| Arhgap24 | 170.0080935 | 2.44E-03 |
| Gm5493 | 169.6740644 | 0.043670587 |
| Skp2 | 169.4493174 | 0.013707985 |
| Txk | 166.3611136 | 0.022464942 |
| Gjb3 | 163.5830543 | 0.014978325 |
| Zfp691 | 162.1746913 | 4.17E-03 |
| Tmem216 | 160.0701744 | 2.21E-06 |
| Hoxc13 | 159.684491 | 0.036161198 |
| Map6 | 158.2246083 | 7.89E-03 |
| Myb | 154.4226862 | 5.42E-06 |
| Gm26767 | 153.3342619 | 0.014518425 |
| Gm7676 | 151.3437949 | 0.01869515 |
| Hoxa9 | 149.857445 | 0.017490432 |
| Marveld2 | 146.1691955 | 0.017825046 |
| Lrrc56 | 145.6857803 | 0.018745712 |
| Prox1 | 144.1198395 | 0.049032125 |
| Cdc6 | 140.9362118 | 2.04E-04 |
| Kbtbd4 | 138.8935225 | 5.62E-04 |
| Itgb2l | 135.227396 | 0.01881671 |
| Mrgpra2a | 134.1275615 | 0.011201369 |
| Trmt13 | 133.1910271 | 0.030355183 |
| Gm2539 | 133.0713173 | 0.034992832 |
| 4632415L05Rik | 131.1797944 | 0.045527996 |
| Slc40a1 | 129.0586238 | 1.99E-03 |
| BC068281 | 125.3081307 | 0.029457581 |
| Trim30b_1 | 124.2042966 | 5.01E-03 |
| C1ql4 | 123.9078333 | 0.02748641 |
| Kcnk12 | 123.6190013 | 0.038339964 |
| Gm20707 | 123.5168229 | 0.018793894 |
| Lonrf1 | 122.0107631 | 0.036054559 |
| Hist1h2bb | 121.9744175 | 0.033310145 |
| Trim30c | 121.3441101 | 0.025396378 |
| 4931406G06Rik | 121.3346583 | 0.024115523 |
| Ly6g | 118.5251534 | 9.19E-03 |
| Tfr2 | 117.8774428 | 9.34E-03 |
| Kif24 | 114.9523042 | 0.027963877 |
| Ccdc84 | 113.9581228 | 0.012467278 |
| Dync2h1 | 113.7975693 | 3.13E-03 |
| Scfd2 | 113.4154575 | 1.09E-03 |
| Zfp809 | 113.234262 | 0.037021406 |
| Gm16433 | 112.2988102 | 0.018693953 |
| 2010002M12Rik | 107.2751369 | 0.026784309 |
| Rab38 | 107.144698 | 1.20E-03 |
| Dnase2b | 107.0921436 | 0.014184558 |
| Hpgds | 106.5460861 | 0.023277023 |
| Gm13822 | 105.6210577 | 0.037219978 |
| Nxpe3 | 102.752706 | 0.0388454 |
| 9830147E19Rik | 101.7821026 | 0.028828264 |
| Clec4f | 99.82949339 | 3.85E-05 |
| Acyp1 | 98.80007424 | 0.022378385 |
| Kifc3 | 96.67472081 | 0.031194507 |
| Rasl11b | 96.65441425 | 6.64E-03 |
| Msrb2 | 94.30797078 | 2.92E-04 |
| Gm14548 | 94.21238582 | 0.022790924 |
| Slc9a9 | 94.02338026 | 0.028290865 |
| Ppp1r9a | 94.02328374 | 0.0283004 |
| Ms4a6b | 90.71031192 | 8.66E-04 |
| Amd-ps4 | 90.45850329 | 0.025082905 |
| Fancm | 89.26047137 | 0.034286656 |
| Dlc1 | 88.70159628 | 5.95E-03 |
| Cma1 | 88.51845392 | 1.32E-03 |
| Npl | 87.23633568 | 7.33E-03 |
| Zfp764 | 84.84807196 | 0.041059896 |
| Zfp251 | 84.82519277 | 0.014915748 |
| Ly96 | 84.75014717 | 0.013265948 |
| Mcm8 | 79.70614372 | 0.047534852 |
| Zfp783 | 78.21263327 | 0.046956346 |
| Spon1 | 77.05954688 | 0.015024055 |
| Dnajc6 | 75.24126691 | 0.048810512 |
| C1qa | 75.0635946 | 5.71E-04 |
| Mfsd6 | 74.73155679 | 0.015049218 |
| I830077J02Rik | 74.50651265 | 1.10E-03 |
| Zc3h12b | 74.33330417 | 0.017763347 |
| Rasgrp2 | 73.61755183 | 3.59E-04 |
| Wdr20a | 73.28019975 | 0.034904304 |
| Elovl3 | 72.55625023 | 0.024713772 |
| Stag3 | 71.75878116 | 0.043370878 |
| Zyg11b | 71.63332267 | 1.11E-04 |
| Klhl36 | 71.04608088 | 1.92E-03 |
| Tlr6 | 70.58837887 | 6.92E-03 |
| Pkd2l1 | 68.1995607 | 0.026879577 |
| Mtcp1_1 | 65.11272567 | 0.047753678 |
| Trim30d | 64.81384813 | 1.74E-03 |
| C230052I12Rik | 62.30157695 | 6.49E-03 |
| Iqgap2 | 61.79117381 | 6.49E-03 |
| Rmdn1 | 61.04509965 | 0.013305527 |
| Klhl15 | 60.67495051 | 0.010575554 |
| Gas2 | 60.15419597 | 6.27E-03 |
| Itgae | 59.49259077 | 0.024397327 |
| Klf5 | 55.36277915 | 0.016459123 |
| Nt5e | 54.68668905 | 1.02E-04 |
| Acsbg1 | 54.08265062 | 0.019913111 |
| Oas2 | 54.05373901 | 8.95E-07 |
| Tmem260 | 53.90413557 | 8.55E-03 |
| B230208H11Rik | 53.42796301 | 8.88E-04 |
| Igha | 51.40834663 | 0.025305537 |
| Cdh17 | 50.98962019 | 0.038723334 |
| Abhd15 | 48.91633224 | 0.029876557 |
| Gm10718 | 48.68514494 | 0.043807027 |
| Bora | 44.20128286 | 7.26E-03 |
| Ifit3 | 44.18064448 | 1.50E-04 |
| 1700020L24Rik | 43.86915248 | 0.018554727 |
| Pafah2 | 43.55258609 | 1.18E-03 |
| Wdr19 | 42.06056388 | 0.046261322 |
| Cars2 | 41.91327947 | 0.026533288 |
| I830012O16Rik | 41.8966228 | 0.014974636 |
| Camp | 41.6506312 | 4.41E-08 |
| Icam2 | 40.98974828 | 6.25E-03 |
| Cpa3 | 40.68178323 | 8.39E-08 |
| Susd1 | 40.53532681 | 9.90E-03 |
| C1qb | 39.70980561 | 2.25E-03 |
| Ifit2 | 39.63183107 | 1.29E-03 |
| Pcyt1b | 39.49155291 | 4.82E-03 |
| Elane | 38.95836108 | 2.61E-07 |
| Nup62 | 38.74798203 | 8.80E-03 |
| Hsd11b1 | 38.35871704 | 7.52E-03 |
| Tab3 | 38.03215567 | 0.019231494 |
| A430105I19Rik | 37.81307585 | 0.032128997 |
| Zfp157 | 36.71135834 | 0.019700132 |
| 2810417H13Rik | 36.60312316 | 0.01266606 |
| Ikzf3 | 36.28214983 | 0.04270645 |
| Pou1f1 | 36.12392734 | 0.03550868 |
| Zfp87 | 36.12297915 | 0.0329979 |
| Mpp7 | 35.90243905 | 0.021044011 |
| Prune2 | 35.82099136 | 0.034700149 |
| 2810006K23Rik | 35.23402265 | 2.92E-03 |
| Slc2a8 | 35.20062225 | 0.010686929 |
| Fam65b | 34.54156698 | 0.014499164 |
| Trim21 | 34.21732114 | 4.67E-03 |
| Rmdn3 | 34.06081946 | 0.022523798 |
| Aldh7a1 | 33.86380613 | 0.023776048 |
| Rbms3 | 33.86017318 | 0.044022756 |
| Clmp | 33.8535337 | 0.018356584 |
| C5ar2 | 33.12709562 | 0.018985571 |
| Cd300ld | 32.91474693 | 2.38E-03 |
| Tjp1 | 32.54251145 | 2.86E-04 |
| Mettl3 | 31.73962161 | 0.015228734 |
| Ankrd13b | 31.34256271 | 0.027174693 |
| Rab37 | 31.30124182 | 0.021256641 |
| Ceacam2 | 31.16498903 | 3.47E-03 |
| Hibch | 30.56562306 | 0.043696975 |
| Zfp871 | 30.22770024 | 0.026096809 |
| Snx24 | 30.03856205 | 3.57E-03 |
| Stfa2 | 29.54742385 | 3.95E-07 |
| Abca13 | 29.54296723 | 1.21E-03 |
| Trmt12 | 29.05458563 | 0.042003283 |
| Phf11a | 28.84446481 | 5.58E-03 |
| Padi4 | 28.19898837 | 3.87E-04 |
| Fam101b | 27.97540811 | 1.73E-03 |
| Nmral1 | 27.4410652 | 9.79E-03 |
| Haus2 | 27.24967368 | 6.35E-03 |
| Trnt1 | 27.10210054 | 0.021066513 |
| Lmnb2 | 26.7536244 | 1.65E-03 |
| Gm21967 | 26.61428869 | 0.041449468 |
| Kif2c | 26.14800272 | 7.19E-03 |
| Tmem238 | 26.07635946 | 5.20E-03 |
| C1qc | 25.36223557 | 1.74E-03 |
| Rhov | 24.90226446 | 0.020261726 |
| Nhsl2 | 24.81762868 | 1.57E-03 |
| Apoe | 24.27631817 | 2.17E-05 |
| Fam149b | 24.18716796 | 0.048443162 |
| Eme1 | 23.80637729 | 5.46E-03 |
| Dhrs13 | 23.27342101 | 0.031811481 |
| Tmem180 | 23.1602227 | 5.79E-03 |
| Ceacam18 | 22.956735 | 0.036418498 |
| Zfp422 | 22.79019524 | 2.64E-03 |
| 4930599N23Rik | 22.45359119 | 0.011596872 |
| Slc24a5 | 22.38724481 | 0.04606763 |
| Apol9b | 21.64622093 | 0.046232857 |
| Nlrc4 | 21.33889389 | 0.018634782 |
| 1700113A16Rik | 21.06480458 | 0.036600297 |
| Mrps22 | 20.79433758 | 0.049249382 |
| Oscp1 | 20.74311939 | 0.049236187 |
| Dok3 | 20.70779287 | 6.03E-04 |
| Spryd7 | 20.03346417 | 0.026465038 |
| Ltf | 19.92168166 | 2.10E-06 |
| Ngp | 19.76815605 | 9.64E-07 |
| Samd5 | 19.34660871 | 0.037566632 |
| Shprh | 19.31057195 | 0.017711961 |
| Stil | 19.07549604 | 3.36E-03 |
| 2010003O02Rik | 19.02483155 | 0.011897319 |
| Slco4a1 | 19.01389717 | 2.18E-03 |
| Dusp12 | 19.00009009 | 0.010688158 |
| Fbxl22 | 18.99114816 | 0.035734875 |
| Tmem156 | 18.94971623 | 0.016762982 |
| 6330418K02Rik | 18.85022708 | 0.030530634 |
| Klf3 | 18.43038585 | 0.036749016 |
| Lenep | 18.02403274 | 0.045625661 |
| Tex2 | 17.93865009 | 3.00E-04 |
| Cd177 | 17.90619442 | 2.33E-04 |
| Klhl23 | 17.44658315 | 0.03008462 |
| Vars2 | 17.24257641 | 0.019494931 |
| BC100530 | 17.05625444 | 2.51E-06 |
| Slfn5 | 16.97075948 | 4.83E-03 |
| Prom1 | 16.96922512 | 0.015454177 |
| Smug1 | 16.93559108 | 0.01027478 |
| Tgfbi | 16.77170033 | 6.72E-05 |
| D16Ertd472e | 16.343816 | 0.032418532 |
| Stfa1 | 16.3218203 | 5.13E-03 |
| Ly6c1 | 16.2525141 | 6.11E-06 |
| Prtn3 | 16.23287329 | 9.18E-06 |
| Syngr1 | 16.17415114 | 0.028821162 |
| Slfn4 | 15.97127612 | 1.31E-05 |
| Ccdc64 | 15.84197628 | 0.032677096 |
| S100a8 | 15.62798081 | 5.80E-06 |
| Fkbp11 | 15.51305022 | 0.029526456 |
| Fcnb | 15.49290007 | 0.013430748 |
| Sipa1l2 | 15.48315659 | 0.022099904 |
| Fdps | 15.29131391 | 1.58E-04 |
| Ebag9 | 15.17898965 | 0.015343534 |
| Smad6 | 15.00229671 | 0.02159914 |
| Snhg3 | 14.99256095 | 1.23E-03 |
| Cd180 | 14.9356192 | 0.027489576 |
| Dennd4a | 14.92785461 | 8.99E-03 |
| Paxip1 | 14.68422442 | 8.19E-03 |
| Ptpla | 14.65378747 | 0.0469611 |
| Kif14 | 14.65255856 | 0.011970127 |
| Frmd4a | 14.52611793 | 2.87E-03 |
| Rab27a | 14.21930452 | 3.42E-03 |
| 9030619P08Rik | 14.11778999 | 2.95E-03 |
| Mdm1 | 14.07148163 | 0.038244437 |
| Ms4a6c | 14.05085349 | 0.021660021 |
| Ddx49 | 14.04043303 | 0.012130134 |
| Nsdhl | 13.51725875 | 0.048965266 |
| Cyp11a1 | 13.47630433 | 3.86E-04 |
| Lipg | 13.24769627 | 6.48E-03 |
| Samd9l | 13.03343529 | 9.49E-05 |
| Dmxl1 | 13.01117817 | 0.032746778 |
| Spink2 | 12.98881323 | 0.015359376 |
| Enpp4 | 12.97858177 | 0.049557362 |
| Trem3 | 12.7580516 | 0.012080565 |
| Specc1 | 12.73971869 | 7.15E-03 |
| Id1 | 12.72524108 | 6.63E-04 |
| Mcm10 | 12.56391522 | 0.014472209 |
| Csf3r | 12.48425031 | 0.026186553 |
| Dennd2a | 12.45857973 | 7.33E-03 |
| Bub1 | 12.39247807 | 6.16E-03 |
| Gp49a | 12.36222292 | 7.42E-03 |
| Armcx3 | 12.17995318 | 0.026688811 |
| Ctsg | 12.15236274 | 5.42E-03 |
| Vamp5 | 12.09678283 | 0.029425966 |
| Polr3f | 12.08625442 | 0.01753301 |
| Capn10 | 12.07909955 | 0.025357512 |
| H6pd | 12.04521099 | 0.012741784 |
| F630028O10Rik | 12.02069831 | 1.92E-03 |
| Stfa2l1 | 11.7137797 | 4.94E-05 |
| Apoc2 | 11.71341927 | 0.029686395 |
| Dgat2 | 11.59232262 | 0.023201626 |
| Gpr97 | 11.58267006 | 1.30E-03 |
| Tnpo3 | 11.5399181 | 0.018234158 |
| Prss57 | 11.53949196 | 0.027949022 |
| Unc5b | 11.36750435 | 0.012118912 |
| 5730508B09Rik | 11.3485536 | 0.015072425 |
| Hmgn2 | 11.25661214 | 1.21E-03 |
| Osbpl11 | 11.16864282 | 0.021767069 |
| Ifitm6 | 11.15267092 | 5.41E-03 |
| Ms4a6d | 11.0406758 | 1.10E-03 |
| Pnpt1 | 10.98340557 | 0.015133549 |
| Tlr3 | 10.93240675 | 0.032331131 |
| Igfbp4 | 10.90292546 | 5.00E-05 |
| Acpp | 10.8649759 | 9.74E-03 |
| Eef2k | 10.70361296 | 0.0278767 |
| Svip | 10.58125387 | 4.37E-03 |
| Oasl1 | 10.50149375 | 2.65E-03 |
| N4bp2 | 10.34190462 | 0.014036927 |
| Gzmb | 10.28709126 | 0.015434528 |
| Mis18a | 10.25769385 | 0.022842699 |
| Mlh1 | 10.2097863 | 0.044720687 |
| Gm5483 | 10.1195182 | 3.42E-05 |
| Cib2 | 10.10219659 | 0.038443078 |
| Epx | 9.991289261 | 1.05E-04 |
| Coq6 | 9.958714029 | 0.04307106 |
| Msl1 | 9.887196991 | 6.54E-03 |
| Isg20 | 9.78198671 | 3.91E-03 |
| Stap1 | 9.779985814 | 0.024042196 |
| Mybl2 | 9.511479709 | 3.83E-03 |
| Plk4 | 9.502785063 | 0.042871908 |
| Mmab | 9.356169416 | 0.026138467 |
| Arhgap1 | 9.316688211 | 3.59E-03 |
| Plac8 | 9.274984157 | 5.01E-04 |
| Dzip3 | 9.269934372 | 0.044690877 |
| Dio2 | 9.217556736 | 0.040296041 |
| Tapt1 | 9.142366841 | 0.042596392 |
| Srm | 9.034049498 | 2.38E-03 |
| Pon3 | 8.989211324 | 0.036546797 |
| Ms4a3 | 8.98712843 | 1.03E-03 |
| Fam219b | 8.960771141 | 0.031306609 |
| Rapgef6 | 8.852812108 | 0.012892619 |
| Rbbp8 | 8.849443919 | 0.018218414 |
| AI607873 | 8.756948995 | 0.031941425 |
| Myl12b | 8.71345672 | 0.028758393 |
| Mapkapk3 | 8.684604417 | 1.35E-03 |
| Ncapg | 8.533959526 | 0.02624934 |
| Gclc | 8.449381307 | 0.020726945 |
| Il1rl1 | 8.440003619 | 0.010085549 |
| Adnp2 | 8.426804919 | 0.035300575 |
| S100a9 | 8.423956524 | 1.26E-04 |
| Il17ra | 8.391664948 | 8.06E-03 |
| Cd33 | 8.147915412 | 0.011796266 |
| Ly6c2 | 8.123623367 | 2.49E-04 |
| Dclre1b | 8.067162132 | 0.042999152 |
| Cd93 | 8.043190059 | 0.018865247 |
| Zfp36l2 | 7.956436321 | 0.019253423 |
| Ing3 | 7.947817434 | 0.03929772 |
| Tal1 | 7.937978828 | 0.017813379 |
| Alox5 | 7.872914352 | 0.012919527 |
| Nceh1 | 7.828127736 | 0.015992615 |
| Rnase4 | 7.786142385 | 0.047719864 |
| Fam107b | 7.709013597 | 2.04E-03 |
| Plscr1 | 7.550126428 | 0.02841106 |
| Ifih1 | 7.532841889 | 6.36E-03 |
| D030056L22Rik | 7.467270034 | 0.024280523 |
| Arhgap4 | 7.46015844 | 0.011606465 |
| Btd | 7.458999079 | 0.028086335 |
| Notch1 | 7.446698896 | 7.84E-03 |
| Ect2 | 7.393952937 | 0.048642035 |
| Rtp4 | 7.385841785 | 2.60E-03 |
| Gm10036 | 7.384873644 | 4.82E-03 |
| Ppp1r3d | 7.381665149 | 0.028653685 |
| Rnaseh1 | 7.366064364 | 0.048801933 |
| Serpinb1a | 7.222795026 | 9.78E-03 |
| Kif11 | 7.219068685 | 0.016210817 |
| Ddx10 | 7.164717624 | 0.013909162 |
| Cpne1 | 7.155634184 | 0.047350801 |
| Fam110a | 7.044948857 | 0.028308874 |
| Prorsd1 | 6.993794528 | 0.017295433 |
| Fcgr4 | 6.922507127 | 0.036146883 |
| Gabbr1 | 6.788548091 | 0.033241144 |
| Plk3 | 6.786215249 | 0.019182224 |
| Hsdl2 | 6.757489003 | 0.044550673 |
| 2310061I04Rik | 6.752682363 | 0.036726901 |
| Naa16 | 6.699283162 | 0.032335945 |
| Ddx60 | 6.672403374 | 2.00E-03 |
| Ilf3 | 6.669124144 | 0.040784707 |
| Lta4h | 6.662073324 | 1.29E-03 |
| Dopey1 | 6.634331157 | 0.047961583 |
| Apobec1 | 6.583962186 | 0.03759426 |
| Cant1 | 6.547752517 | 0.041188205 |
| Cmpk2 | 6.539888595 | 0.011725631 |
| Tmem140 | 6.497463972 | 0.024752281 |
| Znfx1 | 6.406070409 | 0.01310979 |
| Ifit1 | 6.340210596 | 8.63E-03 |
| Cyp1b1 | 6.320359 | 0.029306894 |
| Smchd1 | 6.28837894 | 0.030375746 |
| Syne1_1 | 6.284769889 | 5.50E-03 |
| Inpp5e | 6.231965421 | 0.039047279 |
| Stfa3 | 6.216105863 | 3.12E-03 |
| Cit | 6.143541833 | 0.047365264 |
| Ifitm1 | 6.04221767 | 1.09E-03 |
| D1Ertd622e | 5.921948135 | 0.037606921 |
| Chkb | 5.911088951 | 0.030283617 |
| Arhgap19 | 5.872124846 | 0.049452328 |
| Ifi44 | 5.831648959 | 4.57E-03 |
| Alox5ap | 5.744391884 | 2.65E-03 |
| Nin | 5.648955729 | 0.011790297 |
| Tmem119 | 5.630564707 | 0.049988047 |
| Cklf | 5.604472901 | 0.030043847 |
| Parvg | 5.559742394 | 8.48E-03 |
| Nolc1 | 5.469655759 | 0.01860492 |
| Isg15 | 5.457974401 | 0.024570101 |
| Gcc2 | 5.413915358 | 0.033676365 |
| Cux1 | 5.381660408 | 0.011538194 |
| Ptplb | 5.352695562 | 0.04241655 |
| Cenpf | 5.300613015 | 0.030298162 |
| Lsm6 | 5.175530929 | 0.049159114 |
| Slc36a1 | 5.15838569 | 0.028523202 |
| Daxx | 5.155391819 | 0.01284742 |
| Znrf1 | 5.132902991 | 0.024990862 |
| Rasal3 | 5.061237886 | 0.040795058 |
| Grk6 | 4.91625134 | 0.034188446 |
| Naa40 | 4.908894752 | 0.028663004 |
| Lman1 | 4.89233061 | 0.04717971 |
| Zfp148 | 4.887132856 | 0.033056822 |
| Atp8b4 | 4.848713427 | 8.01E-03 |
| Tgfbr1 | 4.838937694 | 0.019927533 |
| Abr | 4.836966295 | 0.013604525 |
| Sdhc | 4.836283262 | 0.031416555 |
| Tns4 | 4.797865223 | 0.027118844 |
| Gigyf2 | 4.775435934 | 0.04869306 |
| Rsad2 | 4.742203959 | 0.010237461 |
| Chi3l1 | 4.733769582 | 0.030212371 |
| Adpgk | 4.714480563 | 0.02680502 |
| Adcy7 | 4.708589835 | 0.042534963 |
| Clec12a | 4.689685264 | 0.026324806 |
| Emc7 | 4.656330817 | 0.030025333 |
| Mxd1 | 4.647217428 | 0.011291737 |
| Irf7 | 4.59023944 | 8.51E-03 |
| Oas3 | 4.557516097 | 0.036520261 |
| Dnajc13 | 4.525873831 | 0.047280265 |
| Pde7a | 4.512307525 | 0.043729688 |
| Spn | 4.407945671 | 0.01883472 |
| Ccr1 | 4.4074128 | 0.01376713 |
| Pglyrp1 | 4.308154351 | 0.015352191 |
| Clip1 | 4.29534778 | 0.04825589 |
| AB124611 | 4.25061474 | 0.047576647 |
| Ly6a | 4.216013702 | 0.019719126 |
| Sp100 | 4.199072966 | 0.016484989 |
| Uba7 | 4.19399458 | 0.03549002 |
| Tubb4b | 4.163989297 | 0.013923676 |
| Ifi204 | 4.116573606 | 0.032847358 |
| Oasl2 | 4.073963931 | 0.013191041 |
| Dut | 4.057822004 | 0.03158507 |
| Lcn2 | 4.045310076 | 0.01048773 |
| Gda | 4.013812942 | 0.041955591 |
| Mgst2 | 3.958042732 | 0.035913437 |
| Top2a | 3.917890303 | 0.016553581 |
| Tcn2 | 3.910902218 | 0.038134587 |
| Zfhx3 | 3.881745745 | 0.038652157 |
| Prg2 | 3.854748965 | 0.013595367 |
| Tlr2 | 3.808313772 | 0.027250714 |
| mt-Atp8 | 3.775826104 | 0.045467546 |
| Mbd2 | 3.754536005 | 0.027585408 |
| Rnf213 | 3.748131286 | 0.033181113 |
| Furin | 3.629428027 | 0.036454073 |
| Herc6 | 3.592373768 | 0.0335289 |
| Parp14 | 3.578688359 | 0.031145861 |
| Man2b1 | 3.566447439 | 0.024452669 |
| Anxa1 | 3.560294885 | 0.03064164 |
| Ddx58 | 3.533161762 | 0.046792781 |
| Fbxl5 | 3.461340315 | 0.037782989 |
| Lgals3bp | 3.325771682 | 0.039807752 |
| Tyrobp | 3.316476125 | 0.028346626 |
| Cpd | 3.314837076 | 0.032762782 |
| Ubash3b | 3.314639999 | 0.049073347 |
| Hexb | 3.306242894 | 0.034766028 |
| Ffar2 | 3.299369711 | 0.040650708 |
| Dmxl2 | 3.296199119 | 0.048226976 |
| Ifitm3 | 2.913927915 | 0.044919744 |
