# Supplementary material for: Donor myeloid derived suppressor cells (MDSCs) prolong allogeneic cardiac graft survival through programming of recipient myeloid cells in vivo
Source: Sci Rep. 2020 Aug 28;10:14249. doi: 10.1038/s41598-020-71289-z (PMC7455707; doi:10.1038/s41598-020-71289-z)
Supplement: Supplementary file 5 [file 41598_2020_71289_MOESM5_ESM.docx]

Donor Myeloid Derived Suppressor Cells (MDSCs) Prolong Allogeneic Cardiac Graft Survival through programming of Recipient Myeloid Cells *in vivo*

Songjie Cai^1,2,3^, John Y. Choi^1,3^, Thiago J. Borges^1,3^, Hengcheng Zhang^1^, Ji Miao^2^, Takaharu Ichimura^1^, Xiaofei Li^1^, Simiao Xu^2^, Philip Chu^1^, Siawosh K. Eskandari^1^, Hazim Allos^1^, Juliano B. Alhaddad^1^, Saif A. Muhsin^1^, Karim Yatim^1^, Leonardo V. Riella^1^, Peter T. Sage^1^, Anil K. Chandraker^1^, Jamil R. Azzi ^1^

^1^ Transplantation Research Center, Renal Division, Brigham and Women's Hospital, Harvard Medical School, Boston, MA.

^2^ Division of Endocrinology, Boston Children's Hospital, Harvard Medical School, Boston, MA.

^3^ These authors contributed equally to this work.

**Table S4. The mRNA expression in allograft tissue, linked to Figure 2E and Figure S10.**

| *Gene* | cMDC(n=3) | MDSC (n=4) | P value |
| --- | --- | --- | --- |
| Il1a | 22.97 ± 0.6348 | 8.102 ± 1.766 | 0.001 |
| Il1b | 94.19 ± 14.18 | 47.92 ± 9.034 | 0.0339 |
| Il2 | 316.9 ± 67.48 | 81.04 ± 29.92 | 0.0166 |
| Il4 | 1264 ± 343.4 | 168.0 ± 65.15 | 0.0142 |
| Il13 | 264.3 ± 67.71 | 74.43 ± 32.58 | 0.0393 |
| Nlrp3 | 59.24 ± 9.606 | 25.48 ± 6.962 | 0.0325 |
| Ifng | 439.4 ± 16.92 | 157.3 ± 72.04 | 0.0223 |
| Irf1 | 122.7 ± 15.28 | 57.68 ± 16.86 | 0.0406 |
| FoxP3 | 8.33 ± 2.09 | 15.54 ± 1.31 | 0.0072 |
| iNOS | 2194.11 ± 1389.82 | 2845.35 ± 1533.11 | 0.6147 |
| ARG1 | 70.45 ± 24.42 | 57.54 ± 38.46 | 0.7089 |
| Tnf | 137.3 ± 9.665 | 48.04 ± 16.42 | 0.0081 |
